# Rejuvenating aged microglia by p16^ink4a^-siRNA-loaded nanoparticles increases amyloid-β clearance in animal models of Alzheimer’s disease

**DOI:** 10.1186/s13024-024-00715-x

**Published:** 2024-03-16

**Authors:** Hyo Jung Shin, In Soo Kim, Seung Gyu Choi, Kayoung Lee, Hyewon Park, Juhee Shin, Dayoung Kim, Jaewon Beom, Yoon Young Yi, Deepak Prasad Gupta, Gyun Jee Song, Won-Suk Chung, C. Justin Lee, Dong Woon Kim

**Affiliations:** 1 Department of Anatomy and Cell Biology, Chungnam National University College of Medicine, Daejeon, Republic of Korea; 2 Brain Research Institute, Chungnam National University College of Medicine, Daejeon, Republic of Korea; 3 Department of Medical Science, Chungnam National University College of Medicine, Daejeon, Republic of Korea; 4 Department of Pharmacology, Chungnam National University College of Medicine, Daejeon, Republic of Korea; 5 Department of Rehabilitation Medicine, Seoul National University College of Medicine, Seoul National University Bundang Hospital, Seongnam, Republic of Korea; 6 Department of Pediatrics, College of Medicine, Soonchunhyang University Bucheon Hospital, Bucheon, Republic of Korea; 7 Department of Medicine, College of Medicine, Catholic Kwandong University, Gangneung, Gangwon-Do Republic of Korea; 8 Translational Brain Research Center, International St. Mary’s Hospital, Catholic Kwandong University, Incheon, Republic of Korea; 9 Department of Biological Sciences, Korea Advanced Institute of Science and Technology (KAIST), Daejeon, Republic of Korea; 10 Center for Glia–Neuron Interaction, Korea Institute of Science and Technology (KIST), Seoul, Republic of Korea; 11 Center for Cognition and Sociality, Institute for Basic Science, Daejeon, Republic of Korea; 12 Department of Oral Anatomy and Developmental Biology, College of Dentistry Kyung Hee University, Seoul, Republic of Korea

**Keywords:** Alzheimer’s disease, Microglia senescence, Phagocytosis, p16^ink4a^, Cell cycle

## Abstract

Age-dependent accumulation of amyloid plaques in patients with sporadic Alzheimer’s disease (AD) is associated with reduced amyloid clearance. Older microglia have a reduced ability to phagocytose amyloid, so phagocytosis of amyloid plaques by microglia could be regulated to prevent amyloid accumulation. Furthermore, considering the aging-related disruption of cell cycle machinery in old microglia, we hypothesize that regulating their cell cycle could rejuvenate them and enhance their ability to promote more efficient amyloid clearance. First, we used gene ontology analysis of microglia from young and old mice to identify differential expression of cyclin-dependent kinase inhibitor 2A (p16^ink4a^), a cell cycle factor related to aging. We found that p16^ink4a^ expression was increased in microglia near amyloid plaques in brain tissue from patients with AD and 5XFAD mice, a model of AD. In BV2 microglia, small interfering RNA (siRNA)-mediated p16ink4a downregulation transformed microglia with enhanced amyloid phagocytic capacity through regulated the cell cycle and increased cell proliferation. To regulate microglial phagocytosis by gene transduction, we used poly (D,L-lactic-*co*-glycolic acid) (PLGA) nanoparticles, which predominantly target microglia, to deliver the siRNA and to control microglial reactivity. Nanoparticle-based delivery of p16^ink4a^ siRNA reduced amyloid plaque formation and the number of aged microglia surrounding the plaque and reversed learning deterioration and spatial memory deficits. We propose that downregulation of p16^ink4a^ in microglia is a promising strategy for the treatment of Alzheimer’s disease.

**Supplementary Information:**

The online version contains supplementary material available at 10.1186/s13024-024-00715-x.

## Background

Alzheimer’s disease (AD) is a chronic degenerative disorder that causes cognitive decline [1]. AD is an age-related disease characterized by amnesia and is more likely to occur in older adults [2] and is the most common cause of dementia. The main pathological features include the deposition of extracellular amyloid-β (Aβ) plaques within the brain parenchyma [3, 4] and neuronal fiber entanglements [5]. Although several pathophysiological and histological changes occur in AD, an early and characteristic hallmark of the disease in patients with AD and mouse models of AD is the prominent morphological changes, known as reactive changes [6], of glia, microglia, and astrocytes located near Aβ plaques. There has been an increasing focus on the understanding of the microglial contribution to AD pathology and progression, as age-dependent accumulation of Aβ in patients with sporadic AD is associated with reduced Aβ clearance [7, 8].

Microglia are the primary immune-effector cell type in the brain and play an essential role in the maintenance of homeostasis and protection of the brain from infection and insult [9]. Moreover, microglia are responsible for phagocytosis, the process of recognizing, engulfing, and digesting foreign substances, in the central nervous system [10]. In the AD environment, microglia are phenotypically active and form intimate connections with amyloid deposits, extending their processes into the cores of plaques [11]. Specifically, microglia not only recognize the Aβ peptide and initiate an immune response but also migrate to the region of amyloid deposition. Much research has suggested that therapeutic strategies to target microglia may represent an effective approach to prevent AD disease progression. Microglia-mediated damage, rather than pathological tau-induced direct neurotoxicity, was the leading force driving neurodegeneration in a tauopathy mouse model of AD [12].

Although the morphological characteristics of aging microglia are not well established, aged microglia are usually dysplastic with absent, augmented, and twisted cytoplasmic abnormalities [13]. It is presumed that AD pathogenesis exaggerates the morphological changes of microglia caused by aging [14]. After labeling amyloid with Methoxy-XO4, microglial cells containing amyloid were isolated from an AD mouse model and subjected to transcriptomics analysis. This revealed an increase in gene ontology related to ribosome, oxidative phosphorylation, and phagolysosome pathways. Moreover, they demonstrate that microglia without amyloid plaques exhibit reduced levels of active synaptosome phagocytosis. However, functional modulation through Hypoxia Inducible Factor 1 subunit Alpha (HIF1A) or Fibrillar Aβ (fAβ) can enhance synaptosome phagocytosis in vitro. They showed that the transcriptional program associated with XO4^+^ microglia from mice is present in a subset of human microglia isolated from brains of individuals with AD. XO4^−^ microglia displayed transcriptional signatures associated with accelerated ageing, and displayed impaired synapse clearance despite accumulating synaptic material, suggesting a disconnect between phagocytic capacity and cellular state [15]. Therefore, the effect of aging on microglial phagocytosis and Aβ clearance are still unknown, but the ability of aged microglia to digest ingested substances appears to be impaired. Taken together, it is reasonable to suggest that microglial dysfunction originating from aging or amyloid plaque load influences the reactivity of microglia and subsequently dysregulates their ability to take up and degrade Aβ. Important unanswered questions include whether microglial dysfunction in AD is reversible and whether reversal of microglial dysfunction can restore phagocytic capacity to limit amyloid accumulation. Thus, we hypothesized that control of the microglial cell-division cycle could be key to restoring the degraded function of aged microglia. In this pre-clinical study, we aimed to test the relevance of the cellular-senescence risk factor cyclin-dependent kinase (CDK) inhibitor 2A (p16^ink4a^) in the mediation of microglial dysfunction and as a potential target for impairment of phagocytosis and removal of Aβ.

While recombinant viruses such as lentiviruses and adeno-associated viruses (AAVs) have been successfully used to target neurons and astrocytes for transduction, it is well known that microglia remain difficult to transduce [16]. Genetic manipulation using small interfering RNA (siRNA) is frequently used to study genotype–phenotype relationships. siRNA delivery to microglia *in vivo* is difficult because of the high immune reactivity of microglia to transfection agents, perhaps caused by the endogenous phagocytic function of microglia to detect, engulf, and destroy pathogens [17]. Nanoparticles (NPs) have attracted attention as vehicles that improve the therapeutic efficacy of drugs and gene mediators. One such nanomaterial copolymer, poly (D,L-lactic-co-glycolic acid) (PLGA), is one of the most promising drug and gene delivery systems. PLGA is FDA-approved, inexpensive, easy to use, and has increased safety [18]. PLGA NPs have a negative charge and are absorbed into cells by endocytosis. Hence, microglia, renowned for their excellent phagocytic ability, ingest a considerable amount of PLGA NPs. Then, endosomal escape of PLGA NPs is due to a pH change from anionic (at physiological and alkaline pH) to cationic (at endosomal/lysosomal acidic pH), exporting siRNA to the cytosol [19]. Our group previously generated several types of gene regulators encapsulated in PLGA NPs, which successfully targeted microglia to cause their action, prolonging gene-regulatory effects [20–24]. Therefore, we sought to utilize PLGA NPs loaded with p16ink4a siRNA to rejuvenate aged microglia through cell cycle regulation in the 5XFAD mouse model of AD, thereby upregulating phagocytosis by microglial-specific siRNA.

## Materials and methods

### Animals

All study protocols were approved by the Institutional Animal Care and Use Committee of Chungnam National University, Republic of Korea (CNUH-019-A0009-2). Mice were housed in groups on a 12-h light/dark cycle with food and water available. Male and female littermate WT mice and 5XFAD mice with a C57BL/6 J background were used as the control and AD model mice, respectively, and were provided by the Korea Brain Research Institute, Republic of Korea. Mice were genotyped by tail biopsy, tissue digestion, and genomic DNA polymerase chain reaction using the following primers: APP-forward, 5′-AGG ACT GAC CAC TCG ACC AG-3′; APP-reverse, 5′-CGG GGG TCT AGT TCT GCA T-3′; control-forward, 5′-CTA GGC CAC AGA ATT GAA AGA TCT-3′; and control-reverse, 5′-GTA GGT GGA AAT TCT AGC ATC C-3′. All mice were housed under conditions of 40% to 60% humidity at 20 to 26 °C. Ventilation was refreshed 15 to 16 times per hour, lighting was maintained at 150 to 300 lx, and experiments were conducted under the conditions of the Pre-Clinical Experiment Center of Chungnam National University Hospital, Republic of Korea.

### Human brain tissues and ethics approval

Frozen human brain tissues were obtained from the Victorian Brain Bank Network, Australia, supported by The Florey Institute of Neuroscience and Mental Health, The Alfred Hospital, and the Victorian Institute of Forensic Medicine, Australia, and funded by the National Health and Medical Research Council, Australia. Details of the human post-mortem cortex tissues are shown in Suppl. Figure 4A. Human tissue experiments were approved by the Bioethics Committee, Institutional Review Board, Chungnam National University Hospital Industry Foundation, Republic of Korea (Institutional Review Board No. CNUH-2020–10-085).

### Intrathecal injection and intravenous injection

For anesthesia, the mice were administered 5% isoflurane in oxygen within an induction chamber, followed by a transition to a maintenance dose of 2% isoflurane. Each mouse was securely positioned in a stereotaxic frame, immobilized with ear bars, and oriented such that the head formed an approximate 120-degree angle with the body. The NPs were administered directly into the cerebrospinal fluid of the cisterna magna through the atlanto-occipital membrane of the skull and via the C1 vertebra. A Hamilton syringe (26 G needle with a 30° beveled tip) was used to slowly inject by hand 20 µL NPs in phosphate-buffered saline (PBS) over 1 min at a depth of 5 mm. The needle was left in place for an additional minute before removal, and then direct pressure was applied to the puncture site. Furthermore, normal body temperature was sustained consistently throughout the experiment by adjusting the isoflurane dose and utilizing environmental heating [25]. PyrPeg was injected intravenously at 10 mg/kg, and mice were euthanized 2 days later. PyrPeg was a gift from Dr. C. Justin Lee of the Institute for Basic Science, Republic of Korea. PyrPeg images were detected with 450 ~ 650 nm fluorescent wavelengths [26].

### Behavior tests

The Barnes maze test was performed first. The Barnes maze was used to examine hippocampal-dependent spatial learning and memory [27]. This behavioral task is divided into 2 phases: a training phase and a probe test. For the training phase, the mice were placed in the center of the circular platform at the start of each trial and given a defined period of time to find the target escape hole. Affix three distinct shapes (star, square, triangle) onto the wall to indicate the cardinal directions: north, south, east, and west. Additionally, during all training and learning experiments, a metronome sound set at 80 beats per minute (bpm) was employed. If an animal entered the target escape hole before the end of the defined time period, the experiment ended. For each trial, we recorded the time it took for each mouse to find the target escape hole. The mice received 5 trials over 5 days (1 trial per day). On each day (days 1 to 3) of the training phase, the mice were given 2 min of exploration time to help the mouse to find the hidden home cage, which was located through the target escape hole. The mice were also given 5 min per day in the home cage for them to rest and acclimatize.

The radial maze test was performed second and was used to measure spatial working memory [28]. The radial maze was made up of an octagonal central platform (32 cm in diameter) and 8 equally spaced radial arms (each 50 cm long and 12 cm wide). The maze was set in an experimental room with external visual cues. The apparatus was cleaned with ethanol solution between trials. All procedures were performed during the light period of the light/dark cycle. All trials were recorded and analyzed with a Noldus EthoVision XT 15 system, and escape delay, distance traveled, and velocity were measured.

### Mouse brain section preparation and immunofluorescence staining

Mouse brain sections were prepared as previously described [29]. Briefly, mice were transcardially perfused with ice-cold 0.9% saline under deep anesthesia. The mice were then sacrificed, and the whole brain was quickly removed and post-fixed in 4% paraformaldehyde for 1 day and then stored in sucrose (using an increasing gradient of 10%, 20%, and then 30% sucrose). Sagittal Sects. (30 µm) were cut using a cryostat. The collected sections were saved in tissue stock solution for subsequent staining. All brain sections were blocked with 5% normal chicken serum (Vector Laboratories, Inc.) and 0.3% Triton X-100 (Sigma) in cold PBS for 1 h, followed by incubation with these primary antibodies: anti-p16^ink4a^ antibody (1:400; catalog no. MA5-17,142, Thermo Fisher Scientific), anti-β-actin (1:500; catalog no. A5316, MilliporeSigma), anti-amyloid-β (1–42) (1:400; catalog no. ab201060, Abcam), anti-Ki67 antibody (1:200; catalog no. 15580, Abcam), anti-TREM2 antibody (1:200; catalog no. PA5-119,690, Invitrogen), anti-Clec7a antibody (1:200; catalog no. ab217331, Abcam), anti-Lamp1 antibody (1:200; catalog no. ab24170, Abcam), anti-GFAP (1:200; catalog no. MAB360, MilliporeSigma), anti-Iba1 (1:200; catalog no. 019–19741, Wako Pure Chemical Industries, Ltd.), and anti-NeuN (1:200; catalog no. 324307, Cell Signaling Technology). Sections were subsequently washed 3 times with ice-cold PBS at room temperature, followed by incubation with Alexa Fluor 568, Alexa Fluor 488, and Alexa Fluor 647 donkey anti-mouse or donkey anti-rabbit secondary antibodies for 1 h. After another 3 washes in ice-cold PBS, the sections were mounted and coverslipped with mounting solution. Nucleus staining was performed with 4′,6-diamidino-2-phenylindole. Fluorescent images were captured on an LSM 900 confocal laser microscope (Carl ZEISS AG) using 40 × oil immersion with image sizes set at 1024 × 1024 pixels. Density analysis of the captured images was performed using ImageJ software. The images were imported into ImageJ, converted to 8-bit images, and smoothed using a Gaussian filter. To quantify Aβ engulfment by microglia and astrocytes, confocal Z-stacks of mouse brain sections stained for Aβ, Iba1, and GFAP were background-subtracted and smoothed using Imaris software (Oxford Instruments). In addition, the IMARIS software was used to measure the overlapping cell volume for each cell unit and to obtain representative images.

### Cellular senescence assay and 3,3′-diaminobenzidine staining

SA-β-gal staining was performed as described previously and in accordance with the manufacturer’s instructions [30]. Brain sections was incubated overnight at 37 °C without CO_2_ in SA-β-gal detection solution. For 3,3′-diaminobenzidine (DAB) staining, the tissues were exposed to 0.3% hydrogen peroxide in PBS for 10 min to remove endogenous peroxidase activity and then incubated in blocking solution. Next, they were treated with anti-Iba1 antibody overnight at 4 °C followed by incubation with the corresponding biotinylated secondary antibody and streptavidin peroxidase complex (Vector Laboratories, Inc.). Then, the tissues were incubated in a solution of DAB (MilliporeSigma) and hydrogen peroxide, dehydrated, and mounted on glass slides using Permount Mounting Medium (Thermo Fisher Scientific). Sections of cerebral cortex were imaged at the same region for each group using a light microscope (AX70, Olympus). To analyze the association between Iba1 staining and SA-β-gal staining, we performed co-localization analysis using the Scatter J plugin in ImageJ software. In short, the staining image to be analyzed was first divided into channels, and then the brightness values of each channel were mapped to a scatter plot. Also, co-localization analysis is possible through the calculation of the Pearson distribution coefficient provided by the plugin [31, 32].

### Western blotting

Western blotting was performed as previously described [33]. BV2 cells were lysed on ice using radioimmunoprecipitation assay lysis buffer (ATTO Corporation), and the lysate was purified by centrifugation. For brain tissue, mice were killed, and 1 hemisphere was transferred to liquid nitrogen and stored at − 80 ℃. Protein extraction was performed by homogenization in cold PBS with 1% phosphatase inhibitor and 1% protease inhibitor cocktails (Thermo Fisher Scientific) using a homogenizer. Homogenates were extracted in lysis buffer and centrifuged for 20 min at 13,000 rpm. Protein concentration was measured using the Bradford assay. The samples were solubilized in 5 × sodium dodecyl sulfate buffer, boiled for 10 min at 100 °C, and loaded on 12–15% sodium dodecyl sulfate–polyacrylamide gel electrophoresis gels for protein gel electrophoresis. A pre-stained protein standard was used to determine molecular weights. Following electrophoresis, the samples were transferred to nitrocellulose membranes. Nitrocellulose membranes were blocked in 5% non-fat skim milk powder in 1 × Tris-buffered saline with 0.05% Tween 20. The following proteins were analyzed by incubation with primary antibodies. The target proteins were then detected using a chemiluminescent horseradish peroxidase substrate (Thermo Fisher Scientific).

### Quantitative Real-Time PCR

For the RNA extraction assay, BV2 cells were stabilized by seeding 2 × 10^6 cells per well on a dish. The BV2 cells were transfected with p16ink4a siRNA (100 nM) or scrambled (sc) siRNA (100 nM). Lipofectamine 2000 reagent (Thermo Fisher Scientific, Cleveland, OH, USA) was used to transfect the cells according to the manufacturer’s instructions. For tissue, mouse cortex brain was harvested, and the tissue were extracted by homogenizing. Total RNA was isolated using TRIzol reagent following the manufacturer’s protocol (GeneAll, RoboExTM, Thermo Fisher Scientific, Waltham, MA, USA). RNA quantification was performed using a NanoDrop spectrometer (Thermo Fisher Scientific, Waltham, MA, USA). Subsequently, cDNA was prepared from the total RNA using a kit (ENzynomics, Daejeon, Republic of Korea). To determine mRNA levels, qRT-PCR was conducted using the AriaMx Real-Time PCR system, with the expression of GAPDH serving as an internal control. The primers used for PCR were p16^ink4a^ (5’-CCCAACGCCCCGAACT-3’;5-GCAGAAGAGCTGCTACGTGAA-3’), p21 (5-GCAGATCCACAGCGATATCCA-3’; 5-AACAGGTCGGACATCACCAG-3’), IL6 (5-TGAGAAAAGAGTTGTGCAATGG-3’; 5-GGTACTCCAGAAGACCAGAGG-3’), IL1α (5-AGGGAGTCAACTCATTGGCG-3’; 5-TGGCAGAACTGTAGTCTTCGT-3’), IL1β (5-TGCCACCTTTTGACAGTGATG-3’; 5-TGATGTGCTGCTGCGAGATT-3’), Timp1 (5-CACACCAGAGCAGATACCATGA-3’; 5-GGGGAACCCATGAATTTAGCC-3’), Mmp3 (5-GTTGGAGAACATGGAGACTTTGT-3’; 5-CAAGTTCATGAGCAGCAACCA-3’), Mmp12 (5-TGCACTCTGCTGAAAGGAGTCT-3’; 5-GTCATTGGAATTCTGTCCTTTCCA3’), Cxcl1 (5-ACCGAAGTCATAGCCACACTC-3’; 5-CTCCGTTACTTGGGGACACC-3’), Cxcl2 (5’-CCAGACAGAAGTCATAGCCAC-3’; 5-TGGTTCTTCCGTTGAGGGAC-3’), Ccl8 (5-CGGGTGCTGAAAAGCTACGA-3’; 5-TTGGTCTGGAAAACCACAGCTT-3’) and GAPDH (5-CCCTTAAGAGGGATGCTGCC-3’; 5-TACGGCCAAATCCGTTCACA-3’). The ∆∆C_t_ method was employed to assess age-dependent gene expression changes, normalizing the C_t_ values of each sample to a reference gene expression. Amplification of the template was deemed undetectable beyond a threshold of 35 cycles. The minimum expression fold changes for the other age groups were then calculated relative to this threshold.

### Amyloid beta 42 Mouse ELISA assay

Frozen mouse forebrain samples were homogenate as previously described. Samples were centrifuged at 13,000 RPM for 20 min at 4 °C. The supernatant was collected for ELISA. Aβ 1–42 (Thermo Fisher Scientific, Ca # KMB3441) ELISAs were performed according to the manufacturer’s instructions [34]. Aβ 1–42 was measured using Sunrise microplate reader (Tecan Group Ltd.) at 450 nm.

### Poly (D,L-lactic-*co*-glycolic acid) nanoparticle production and characterization

All reagents and solvents were of analytical grade. The PLGA (Purasorb® PDLG 5002A, Corbion N.V.) NPs were prepared with minor modifications, as previously reported [35]. All types of PLGA NPs were purchased from Nanoglia (Daejeon, Republic of Korea) [25]. To prepare the siRNA-encapsulated PLGA NPs, 20 μmol of each siRNA (Invitrogen; Ca# 53,640, The sequence of p16^ink4a^ siRNA as follow; sense 5’-GGUGAUGAUGAUGGGCAACtt-3’, antisense 5’-GUUGCCCAUCAUCACCtg-3’) in 200 μL autoclaved distilled water was added dropwise to 0.8 mL dichloromethane containing 2.5 mg PLGA and emulsified by sonication (10% of maximum frequency for 30 s; SFX 550, Branson Ultrasonics) to form a primary W1/O emulsion. Next, 2 mL 1% (w/v) PVA1500 (Alfa Aesar) was added, and the mixture was further emulsified by sonication for 1 min to form a W1/O/W2 double emulsion. Then, 6 mL 1% (w/v) PVA1500 was added, and the dichloromethane was evaporated by magnetic stirring for 3 h at room temperature in a fume hood. Finally, the PLGA NPs were collected by centrifugation at 13,000 rpm for 10 min at 4 °C, washed twice with deionized water, and freeze-dried [21, 36].

PLGA-rhodamine conjugated NPs were prepared by mixing PLGA and PLGA-rhodamine B endcapping (AV027, PolySciTech, Akina, Inc.). Nanoparticles were manufactured by mixing two types of polymers in a ratio of 9 to 1. AAV-GFP-encapsulated PLGA NPs were prepared by mixing 100 µg AAV-GFP plasmid and PLGA NPs. The NPs were diluted in double-distilled water to analyze their size and zeta potential by a dynamic light-scattering assay using the Zetasizer Nano ZS90 (Malvern Instruments), as previously described [23]. Scanning electron micrographs of the NPs were obtained with a scanning electron microscope (SNE-4500 M, SEC Co. Ltd.).

### siRNA loading efficiency and release assay

The siRNA loading efficiency was measured using 5 mg/mL NP powder in dimethyl sulfoxide. Loading efficiency (%) = (released siRNA after complete hydrolysis/total amount of NPs) × 100. The siRNA release assay was performed by incubation of NP powder loaded with siRNA in 250 µL PBS. The supernatant was collected continuously for 5 days, and the siRNA concentration was calculated using a Nanodrop spectrometer (Thermo Fisher Scientific).

### BV2 cell culture, transfection and cell viability assay

The BV2 immortalized murine microglial cell line was received from Dr. Eun-Hye Joe of Ajou University School of Medicine, Suwon, Republic of Korea [4]. BV2 cells were grown in Dulbecco’s modified Eagle medium with 10% fetal bovine serum. For siRNA transfection, BV2 cells were plated in 6-well plates and cultured to approximately 60% confluency when cells were added. BV2 cells were transfected with p16^ink4a^ or scrambled siRNA with Lipofectamine 2000 (Thermo Fisher Scientific) in accordance with the manufacturer’s instructions. To determine the cytotoxicity of siRNA-encapsulated PLGA NPs, cell viability was measured using the EZ-Cytox cell viability assay kit (Daeil Lab Service Co. Ltd.) in accordance with the manufacturer’s instructions. Cell viability was quantified by measuring the absorbance at 540 nm using a Sunrise microplate reader (Tecan Group Ltd.).

### Phagocytosis assay and fluorescence-activated cell sorting

Aβ (1–42)-conjugated pHrodo was a gift from Dr. Won-Suk Chung of the Korea Advanced Institute of Science and Technology, Republic of Korea. BV2 cells were treated with Aβ-pHrodo for 3 h. To visualize lysosomes in live cells, BV2 cells were stained with 0.5 µM LysoTracker Green DND-26 (Thermo Fisher Scientific) for 3 h at 37 °C in a humidified 5% CO_2_ incubator. Live cell imaging was performed with an LSM 900 confocal laser scanning microscope.

BV2 cells were transfected with p16^ink4a^ or scrambled siRNA for 48 h. To measure phagocytosis by fluorescence microscopy, BV2 cells were treated with 0.1 mg/mL pHrodo Red Zymosan Bioparticles Conjugate (P35364, Thermo Fisher Scientific) for 3 h. BV2 cells were washed briefly in PBS and then nuclear DNA was labeled with 4′,6-diamidino-2-phenylindole. For FACS analysis, the cells were stained with the pHrodo bioparticle conjugates for 3 h, washed in PBS, and briefly trypsinized to detach the cells. BV2 cells were then resuspended in 300 µL PBS, and FACS analysis was performed using a BD FACSCanto II flow cytometer (BD Biosciences). Apoptosis was measured by flow cytometry using the EzWay Annexin V-FITC Apoptosis Detection Kit (K29100, Koma Biotech) in accordance with the manufacturer’s instructions. Briefly, the cells were dissociated with trypsin, fixed in 4% paraformaldehyde at − 20 °C, and propidium iodide was used to determine the cell cycle distribution. After staining with annexin V-FITC, the cellular DNA content was evaluated on a BD FACSCanto II flow cytometer. For BrdU labeling and detection, BV2 cells were treated with 10 μM BrdU for 3 h. The existing culture medium was then removed and replaced with new culture medium for a 1-day incubation period. After that fix and permeabilized cells according to standard immunocytocyemistry with anti-BrdU antibody (1:200; catalog no. ab220076, Abcam).

### Data acquisition and bioinformatics analysis

We searched the Gene Expression Omnibus (https://www.ncbi.nlm.nih.gov/geo/) and GTEx Portal (https://gtexportal.org/) to investigate transcriptomic alterations in the aged brain. First, we gratefully downloaded the RNAseq data set GSE205803, which the authors gladly provided [37]. Briefly, the authors treated enriched microglia isolated from young mice (young group, < 3 months old, *n* = 4) and aged mice (old group, > 18 months old, *n* = 4) with 2.5 μM fluorescently labeled Aβ (1–42) for 2 h. The microglia were divided into 2 groups by FACS (those that took up Aβ42 and those that did not) and were subjected to bulk RNA sequencing [37]. The downloaded data values were already normalized as transcripts per million (TPM), and we compared the gene expression of Aβ42-positive microglia obtained from the young and old groups using t tests. Gene ontology enrichment analyses were performed with significantly altered genes with P values less than 0.01 using PANTHER (version 16.0) [38]. The genes classified as replicative senescence, GO0090399, were depicted using Z-scores transformed from TPM scales. Second, we downloaded the subject phenotypes and gene TPM V8 data of brain tissues from the GTEx Portal. *CDKN2A* expression was classified in accordance with brain anatomy and compared by subject age using the Kruskal–Wallis test and the Dunn multiple comparisons test by GraphPad Prism.

### Illustrations

Illustrations were generated using Biorender (http://www.biorender.com).

### Statistical analysis

Statistical analyses were performed with GraphPad Prism version 9.0. The statistical differences between 2 groups were determined by unpaired Student’s t tests. Data were analyzed by repeated measures 1-way analysis of variance (ANOVA) with post hoc Bonferroni tests or by repeated measures 2-way ANOVA with Tukey multiple comparison tests. All data are expressed as mean ± standard error of the mean (SEM). A value of *p* < 0.05 was considered to be statistically significant (Suppl. Table 1).

## Results

### Microglial cells are activated near and co-localize with amyloid-β plaques in the 5XFAD mouse model of Alzheimer’s disease

We used 5XFAD mice, a representative animal model of AD, and their wild-type (WT) control littermates that lack Aβ accumulation. We observed mild to moderate Aβ deposition in the brains of 5XFAD mice at 4 months of age and heavy Aβ deposition in the brain and dementia symptoms at 8 months of age. Amyloid plaques formed throughout the brains of 5XFAD mice from 4 months of age, including the hippocampus and prefrontal cortex (Suppl. Figure 1A). Glial cells were activated around these amyloid plaques, as is well known [39]. The number of Iba1- and GFAP-immunoreactive cells (to identify the activated forms of microglia and astrocytes, respectively) was increased in the hippocampus (Suppl. Figure 1B) and cortex (Suppl. Figure 1C) around Aβ plaques in 8-month-old 5XFAD mice. Expression of Aβ was markedly increased in 8-month-old 5XFAD mice compared with their age-matched control littermates. Iba1 and GFAP expression were increased in the cortex of 8-month-old 5XFAD mice, whereas the expression of the neuronal marker NeuN was reduced (Suppl. Figure 1D, E).

**Fig. 1:**
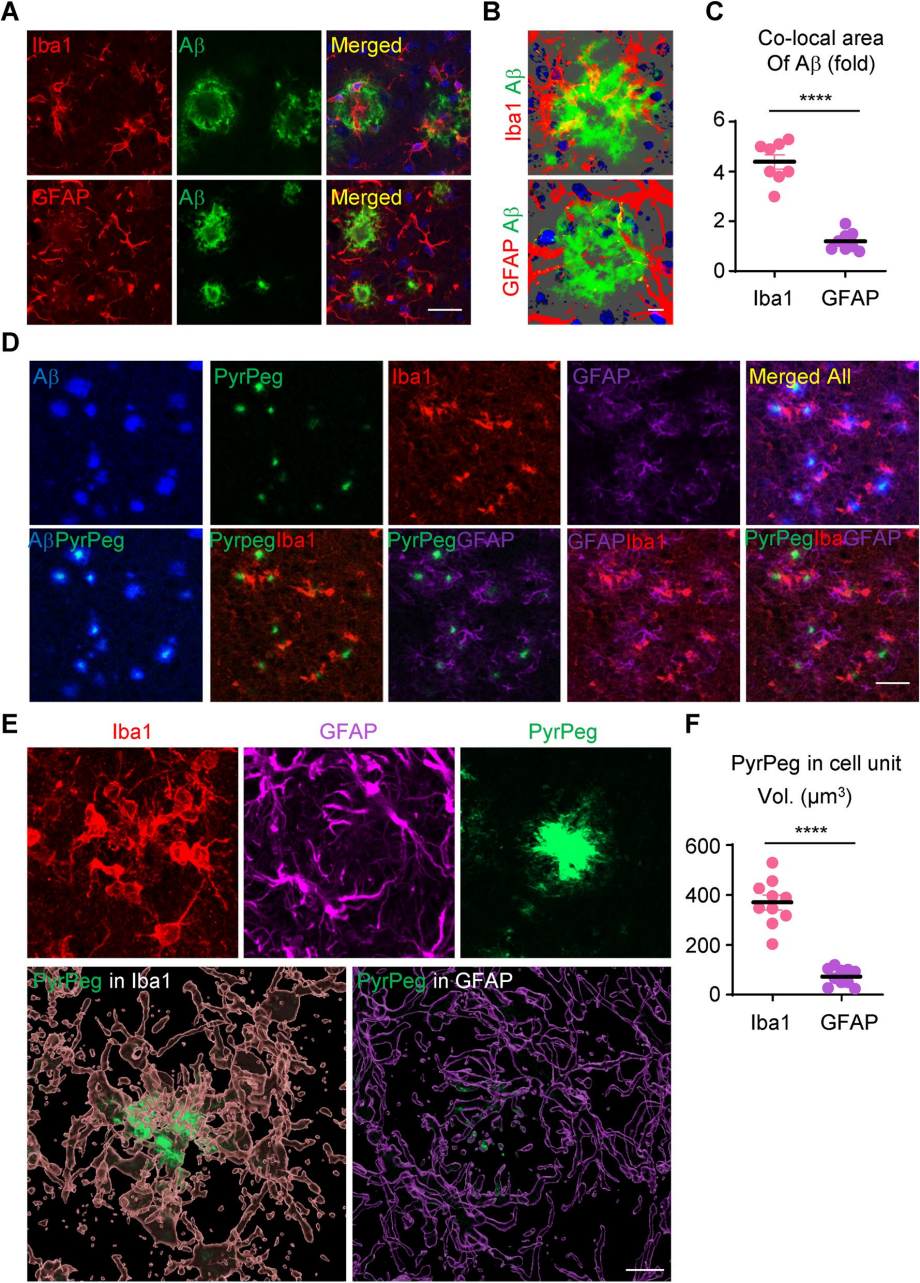
Microglia that contain aggregated amyloid-β are activated and are located near neuritic plaques in 5XFAD mice. **A** Z-stack images of brain tissue from 5XFAD mice after staining with Iba1 or GFAP in the peri-plaque region. Scale bar: 200 µm. **B** Three-dimensional reconstruction of Z-stack images in which Aβ overlaps with staining by anti-Iba1 antibody or anti-GFAP antibody. Scale bar: 10 µm. **C** Quantification of Aβ volumes co-localized with glial volumes, as measured in cell units. ****p < 0.001, Aβ volume in Iba1 volume versus Aβ volume in GFAP volume; unpaired Student’s t test. **D** Representative images of 5XFAD mouse brain sections stained with an anti-Aβ antibody (blue), PyrPeg (green), anti-Iba1 antibody (red), and anti-GFAP antibody (magenta). Scale bar: 100 µm. **E** Orthogonal view of a 3-dimensional reconstruction of confocal images showing co-localization of an Aβ plaque (green) with Iba1(pink) or GFAP (purple). Scale bar: 10 µm. **F** Quantification of PyrPeg co-localized with microglia versus astrocytes, as measured in cell units. ****p < 0.001, PyrPeg volume in Iba1 volume versus PyrPeg volume in GFAP volume; unpaired Student’s t test

Morphological changes of glial cells are well-known, early, and characteristic features in patients with AD and mouse models of AD [6, 40]. The microglia and astrocytes that surround Aβ plaques undergo a marked morphological change known as gliosis. We found that microglia were more centrally located near the Aβ plaques of 5XFAD mice than were astrocytes (Fig. 1A). To understand the association of microglia with the growth pattern of individual Aβ plaques, we performed regression analysis for the number of surrounding Iba1-positive cells with Aβ plaque size in the brains of 8-month-old 5XFAD mice. Surprisingly, Aβ clusters were associated with a greater number of microglia as the size of the plaques increased (Suppl. Figure 2A). Suppl. Figure 2B shows the correlation between Aβ plaque size and Iba1-positive cell numbers, based on measurements of representative images in Suppl. Figure 2A. Pearson analysis revealed a significant correlation between Aβ plaque size and Iba1-positive cell numbers (*r* = 0.9964, *p* < 0.0001, *n* = 210). For subsequent analysis, we used data from plaques larger than 200 µm in diameter, because the larger plaques correlated with an increased number of nearby activated microglia.

There are several hypotheses about the influence of astrocytes and microglia on amyloid plaques [41]. To find out if microglia or astrocytes have a greater effect on peri-plaque complexity, we quantified confocal microscope Z-stack images. We imaged 15 µm of tissue depth, performed 3-dimensional reconstruction, and found that the area of co-localization with dendrites and amyloid plaques for microglia was greater than 4.3-fold more than for astrocytes (Fig. 1B, C). Specifically, Aβ plaques co-localized with microglia had a greater number of branches and a greater area of plaques and processes compared with plaques co-localized with astrocytes.

PyrPeg, a novel two-photon fluorescent probe that selectively targets insoluble Aβ, has been proven to detect neuritic plaques and not tau aggregates [26]. Intravenous injection of PyrPeg allows detection of neuritic plaques in the brains of mouse models of AD. We used PyrPeg to specifically stain the cores of neuritic plaques and tested the co-localization of microglia, astrocytes, and Aβ (Fig. 1D). Interestingly, we observed that the volume of PyrPeg co-localized with Iba1-positive cells was greater than with GFAP-positive cells (Fig. 1E, F). These data confirm that microglia are an important potential contributor to Aβ plaque deposition and progression in brains affected by AD.

### P16^ink4a^ expression increases around amyloid plaques and in microglia in postmortem brains of patients with Alzheimer’s disease and 5XFAD mice.

**Fig. 2:**
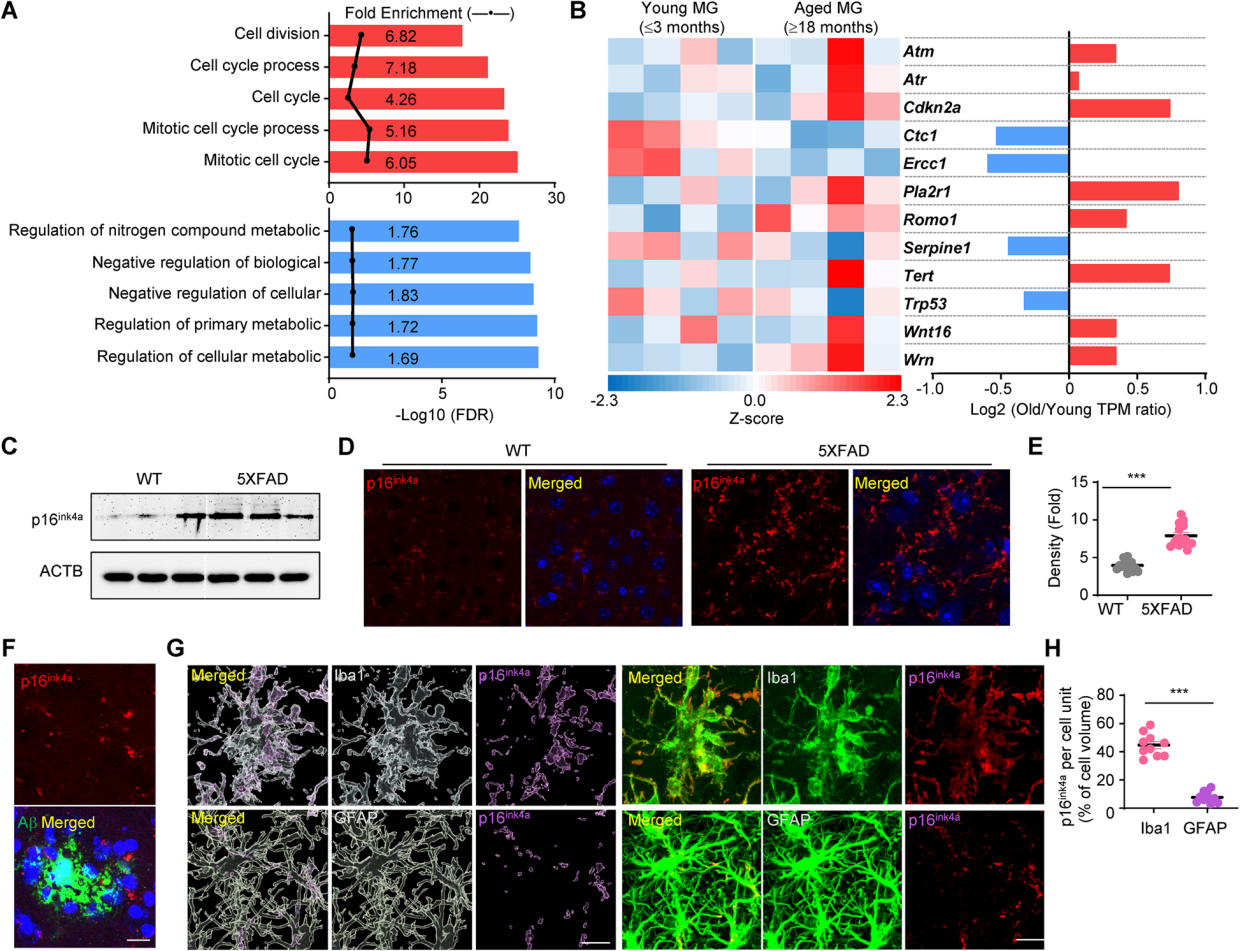
*Cdkn2a* and its coding protein p16^ink4a^ are increased in senescent microglia, but not astrocytes or neurons, in postmortem brains of patients with Alzheimer’s disease and 8-month-old 5XFAD mice. **A** Top 5 gene ontology biological process pathways involved in Aβ42-positive microglia from old mice compared with young mice. The upper panel (red) and lower panel (blue) show the pathways enriched by increased and decreased gene expression, respectively. FDR: false discovery rate. **B** Heat map representing gene expression relating to replicative senescence (GO0090399) in Aβ42-positive microglia (MG) from young and old mice (*n* = 4 for each group). The scale represents the Z-score from 2.3 (highest expression) to − 2.3 (lowest expression). The bar graph reveals the ratio of the gene expression levels in Aβ42-positive microglia between young and old mice. **C** Western blot of the protein levels of p16^ink4a^ in brain tissue from WT and 5XFAD mice. ACTB: β-actin. **D** Immunohistochemistry of p16^ink4a^ and DAPI staining in brain tissue from WT and 5XFAD mice. Scale bar: 100 µm. **E** Quantification of the protein levels in (**D**). ***p < 0.005, 5XFAD versus WT; unpaired Student’s t test. **F** Z-stack confocal images of p16^ink4a^ (red) with Aβ plaque (green). Scale bar: 20 µm. **G** Representative images show p16^ink4a^ expression (purple) with Iba1 or GFAP immunostaining. Representative images were constructed using Imaris software. Scale bar: 20 µm. **H** The percentage of p16^ink4a^ expression per cell volume. ***p < 0.005, Iba1 versus GFAP; unpaired Student’s t test

To investigate the characteristics of microglia in aging, we analyzed the GSE205803 transcriptomic data of Aβ42-positive microglia isolated from young and old mice (see Methods). We generated a list of differentially expressed genes (DEGs) in the Aβ42-positive microglia from old mice compared with young mice and performed gene ontology enrichment analysis with 418 increased DEGs and 375 decreased DEGs to gain insight into the underlying biology of aged microglia. DEGs that were increased in Aβ42-positive microglia from old mice were enriched in cell cycle process (GO0022402), cell cycle (GO0007049), mitotic cell cycle process (GO1903047), and mitotic cell cycle (GO0000278) (Fig. 2A upper panel, Supple. Table 2). DEGs that were decreased in Aβ42-positive microglia from old mice were enriched in regulation of nitrogen compound metabolic process (GO0051171), negative regulation of biological process (GO0048519), negative regulation of cellular process (GO0048523), regulation of the primary metabolic process (GO0080090), and regulation of cellular metabolic process (GO0031323) (Fig. 2A lower panel, Supple. Table 2). As the mitotic cell cycle pathways were significantly involved, we investigated the expression of replicative senescence-related genes. *Cdkn2a*, *Pla2r1*, and *Tert* were increased and *Ercc1* was decreased by over 50% in Aβ42-positive microglia from old mice (Fig. 2B).

*Cdkn2a*, also known as CDK inhibitor 2A, encodes 2 proteins, including the INK4a family member p16 (p16^ink4a^) and p14arf. P16^ink4a^, a representative factor of aging, has increased expression in the tissues of patients with AD [42]. First, we evaluated *CDKN2A* expression in the human brain using Genotype-Tissue Expression (GTEx) data (see Methods). Interestingly, AD-associated brain regions, such as the cortex, frontal cortex Brodmann area 9, and hippocampus, had increased *CDKN2A* expression with aging (Suppl. Figure 3). Next, because we expected that p16^ink4a^ expression affects the formation of amyloid plaques by influencing microglial aging, we tested whether p16^ink4a^ expression was increased in brain tissue from patients with AD (Suppl. Figure 4A, B). The p16^ink4a^ protein levels in the cortex of patients with AD were investigated and compared with those in the cortex of age-matched controls. Western blotting was performed using post-mortem cortex tissues from the Victorian Brain Bank Network, Australia. The p16^ink4a^ protein levels were significantly increased in the cortex of patients with AD compared with those in the cortex of age-matched controls (Suppl. Figure 4C). The protein expression level of supply. Figure 4C was quantified and graphed (Suppl. Figure 4D). The results presented in Suppl. Figure 4D also revealed an increase in the level of Aβ and Iba1 as evidence of microglial reactivity. Then, we performed co-immunofluorescence staining using an anti-p16^ink4a^ antibody together with an anti-Iba1 antibody to assess the localization of p16^ink4a^ in the cortex of patients with AD. We observed significant co-localization of p16^ink4a^ expression with Iba1 in the cortex of patients with AD (Suppl. Figure 4E).

Similarly, we showed that the expression of p16^ink4a^ was also increased in the 5XFAD mouse model (Fig. 2C). The increase in expression of p16^ink4a^ in the tissues of 5XFAD mice older than 8 months of age was approximately twofold higher than in WT mice (Fig. 2D, E). As we expected, increased expression of p16^ink4a^ was observed more often around amyloid plaques in 5XFAD mice (Fig. 2F). Immunostaining for Iba1 and GFAP showed that p16^ink4a^ expression co-localized with microglia (Fig. 2G), and p16^ink4a^ expression was detected in the majority of microglia in the cortex and hippocampus near Aβ plaques in 5XFAD mice (Fig. 2H). Based on these results, the control of microglial p16^ink4a^ expression is expected to be a useful approach to slow the progression of AD.

### P16^ink4a^ siRNA-loaded poly(D,L-lactic-co-glycolic acid) nanoparticle characterization and delivery to microglia in vitro and in vivo

**Fig. 3:**
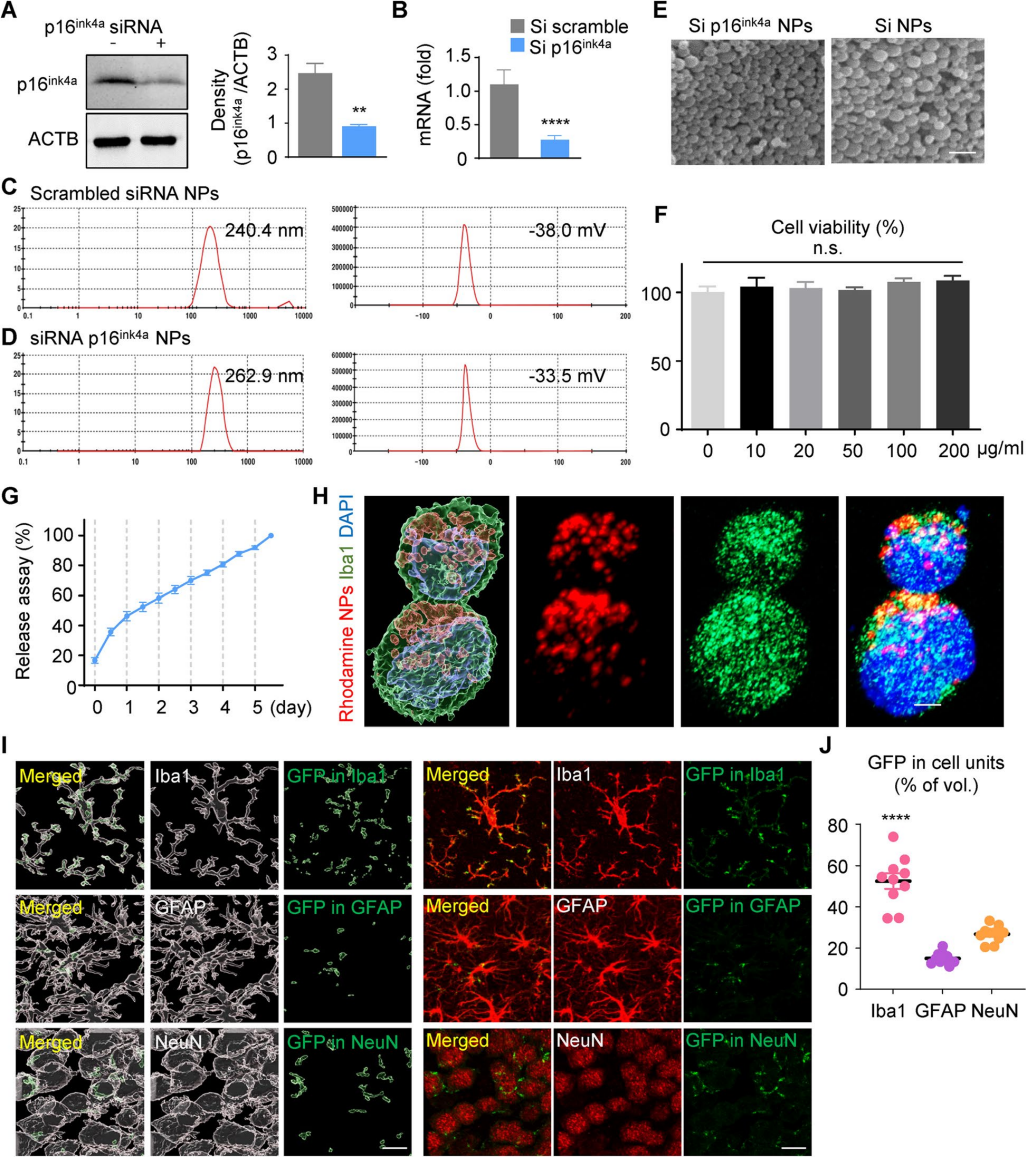
Characterization and morphology analysis of poly(D,L-lactic-co-glycolic acid) nanoparticles with p16^ink4a^ siRNA or scrambled siRNA. **A** Western blot and quantification of the protein levels of p16^ink4a^ after siRNA transfection in BV2 cells. **p < 0.01, versus scrambled siRNA control; unpaired Student’s t test. ACTB: β-actin. **B** mRNA expression of p16^ink4a^ was quantified by qPCR after transfection in BV2 cells. The data are expressed as the mean ± SEM. ****p < 0.001, versus scrambled siRNA control, unpaired Student’s t test. **C**,**D** The size and zeta potential of NPs containing scrambled siRNA (Si) (**C**) and p16^ink4a^ siRNA (**D**). **E** Scanning electron microscope images of NPs. Scale bar: 300 nm. Original magnification: 15,000 × . **F** MTT cytotoxicity assay of BV2 cells incubated with p16^ink4a^ siRNA encapsulated PLGA NPs (0–200 µg/mL) for 24 h. Data are expressed as mean ± SEM (*n* = 4) and were analyzed by 1-way ANOVA followed by the Tukey test for multiple comparisons. n.s.: not significant. **G** The encapsulation efficiency of siRNA NPs as measured by the release of siRNA over 5 days by Nanodrop spectrophotometer. We achieved encapsulation of 31.8% ± 0.1% (mean ± SEM) of the total siRNA into PLGA NPs. **H** BV2 cells immunostained with anti-Iba1 antibody and 4′,6-diamidino-2-phenylindole (DAPI) after treatment with rhodamine-tagged PLGA NPs for 3 h. Representative images were constructed using Imaris software. Scale bar: 10 µm. **I** Immunostaining of brain tissues 3 days after injection of AAV-GFP plasmid-loaded PLGA NPs. Tissues were stained with antibodies to Iba1 (microglia marker), GFAP (astrocyte marker), and NeuN (neuronal marker) to visualize the distribution of GFP in the cortex. Representative images were constructed with Imaris software, and GFP fluorescence merged into each cell-type marker was expressed by image sorting. Scale bar: 20 µm. **J** The volume of merged GFP for each cell-type marker was graphed as a percentage of the total GFP volume. Data are expressed as the mean ± SEM (*n* = 10 for each group) and were analyzed by 1-way ANOVA followed by the Tukey test for multiple comparisons. ****p < 0.001, Iba1 versus GFAP, and Iba1 versus NeuN

We selected PLGA NPs to test the effects of a reduction in p16ink4a expression in 5XFAD mice. The PLGA polymer is safe and has been approved by the United States Food and Drug Administration. Our previously published study reported that it selectively targets microglia [21–23]. First, we checked the effect of the p16ink4a blockade by siRNA in microglial cells. The BV2 cells were transfected with p16ink4a siRNA (100 nM) for 2 days. The scrambled siRNA (100 nM) was used as the negative control. We confirmed that p16^ink4a^ expression was decreased more than threefold by p16^ink4a^ siRNA in BV2 microglial cells (Fig. 3A). Also, mRNA level of p16ink4a reduced relative to the scrambled siRNA transfected cells by qPCR (Fig. 3B). We loaded the NPs with 20 µM scrambled siRNA or p16^ink4a^ siRNA. We measured the average size and zeta potential of the scrambled siRNA NPs (240.4 ± 58.49 nm and − 38.0 ± 6.68 mV, respectively; Fig. 3C) and the p16^ink4a^ siRNA NPs (262.9 ± 66.88 nm and − 33.5 ± 5.68 mV, respectively; Fig. 3D). We confirmed the spherical nature of PLGA NPs by scanning electron microscopy (Fig. 3E). We tested the biocompatibility and stability of PLGA NPs and found there was no significant change in BV2 cell viability after treatment with a range of NP doses (Fig. 3F). We analyzed the siRNA encapsulation efficiency and release profiles of p16^ink4a^ siRNA NPs using a Nanodrop spectrophotometer 3 times per day from day 1 to day 5. The encapsulation efficiency was 31.8% ± 0.1% (Fig. 3G). The highest release rate was maintained from day 1 to day 5 of hydrolysis at pH 7.4.

Next, we measured PLGA NP uptake by BV2 cells. Because siRNA-loaded PLGA NPs do not fluoresce, rhodamine-conjugated PLGA (rho-PLGA) NPs that emit red fluorescence were prepared to trace their cellular uptake in real-time. Most BV2 cells have a round-form, and Rhodamine fluorescence was observed in the cytosol, clearly distinguishable from the nucleus. Endocytosis of rho-PLGA NPs was observed in BV2 cells using Imaris microscopy image analysis software (Fig. 3H). Then, to confirm the cell-type specificity of the NPs in the brain, green fluorescent protein (GFP)-expressing AAV (AAV-GFP) vectors were synthesized, encapsulated by PLGA NPs, and injected into the brains of WT mice intrathecally via the cisterna magna. After 3 days, the mice were euthanized, brain tissues were extracted, and the green fluorescence in each cell unit was imaged separately using Imaris software (Fig. 3I). The volume of green fluorescence was quantified, and the greatest amount of green fluorescence co-localized with microglial locations (Fig. 3J). Taken together, these data suggest that p16^ink4a^ siRNA loaded PLGA NPs can be used to effectively deliver drugs to microglia.

### Reduction of p16^ink4a^ expression in microglia using p16^ink4a^ siRNA-loaded nanoparticles reverses spatial memory and learning deficits in 5XFAD mice.

**Fig. 4:**
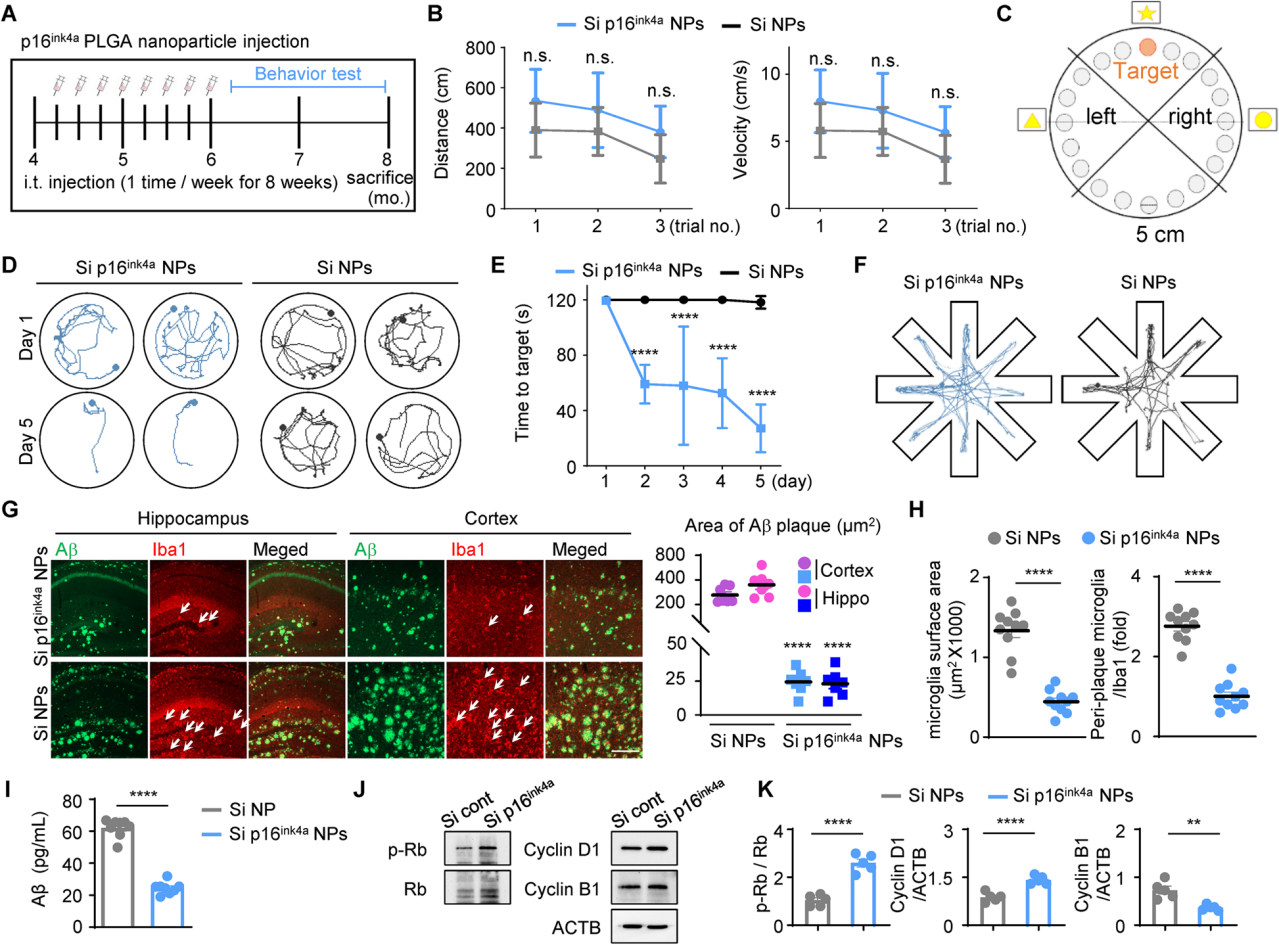
Microglia-specific p16^ink4a^ downregulation protects against deterioration of spatial memory and learning in the 5XFAD mouse model of Alzheimer’s disease and promotes the division of microglia, which interferes with cellular senescence. **A** Schematic of the experimental design for PLGA NP injection into 5XFAD mice. i.t.: intrathecal. **B** Distance and velocity graphs from the Barnes maze test for mice injected with p16^ink4a^ siRNA NPs or scrambled siRNA NPs. Data are expressed as mean ± SEM (*n* = 7) of 3 trials and were analyzed by 2-way ANOVA followed by the Bonferroni test for multiple comparisons. n.s.: not significant. **C** Schematic of the experimental design for the Barnes maze test. **D** Representative movement paths of mice in the probe test of the Barnes maze test 24 h after the last training day. **E** The time taken for mice to reach the target in the Barnes maze test over 5 training days. Data are expressed as mean ± SEM (*n* = 8) and were analyzed by 2-way ANOVA followed by the Bonferroni test for multiple comparisons. ****p < 0.001 **F** Representative searching tracks of mice injected with p16^ink4a^ NPs or scrambled siRNA NPs in the radial maze test. **G** In both cortex and hippocampus, Aβ and Iba1 immunostaining paralleled a decrease in the area covered by Aβ plaques in mice injected with p16^ink4a^ siRNA NPs. Scale bar: 100 µm. Data are expressed as mean ± SEM (*n* = 7) and were analyzed by 1-way ANOVA followed by the Tukey test for multiple comparisons. ****p < 0.001 **H** The surface area of microglia and the number of microglia around the plaque were measured. White arrows indicate masses of 3 or more microglia around the plaque. ****p < 0.001, versus scrambled siRNA NPs; unpaired Student’s t test. **I** ELISA of cortex lysate showed decreased of Aβ1-42 of mice infected with PLGA NPs. Each protein lysate samples from cortex were measured based on 50 μg by bradford assay. ****p < 0.001, versus scrambled siRNA NPs; unpaired Student’s t test. **J** Western blot of p-Rb, Rb, cyclin D1, and cyclin B1 expression in cortex tissue of mice injected with PLGA NPs. β-Actin (ACTB) was used as a protein loading control. **K** Quantification of the protein levels in (**J**) after injection of p16^ink4a^ siRNA PLGA NPs in 5XFAD mice brain tissue. **p < 0.01 and ****p < 0.001, versus scrambled siRNA PLGA NPs; unpaired Student’s t test

We aimed to verify the effects of downregulation of p16^ink4a^ expression on the progression of AD. We injected p16^ink4a^ siRNA NPs or scrambled siRNA NPs into the cisterna magna of 4-month-old 5XFAD mice once per week for a total of 8 weeks (Fig. 4A). We examined behavioral changes in the mice injected with p16^ink4a^ siRNA-loaded NPs or scrambled siRNA NPs 1 to 2 weeks before euthanasia and then obtained the tissues for molecular and histological analysis. At 8 months of age (i.e., 2 months from the last NP injection), p16^ink4a^ siRNA NP-injected mice had significantly reduced p16^ink4a^ mRNA level (Suppl. Figure 5A) and protein levels (Suppl. Figure 5B, C) and p16^ink4a^ immunoreactivity (Suppl. Figure 5D, 5E) in the brain cortex compared with mice treated with scrambled siRNA NPs. There was no effect on mouse weight or external phenotypic features between the 2 groups (data not shown). We investigated the effect of p16^ink4a^ siRNA NPs on motor dysfunction and ataxia. It was confirmed that there was no significant difference in distance traveled and velocity between the 2 groups (Fig. 4B), indicating that there is no association between the expression level of p16^ink4a^ and motor function.

Learning and memory impairments were quantified using the Barnes maze and the radial maze behavioral tests. The Barnes maze test measures the time to locate a target on a circular disk, with 2-min trials performed at similar times each day for 5 days (Fig. 4C). The time to locate the target was similar for both groups of mice on the 1st day; the time to locate the target was significantly faster in the p16^ink4a^ siRNA NP-treated group from the 2nd day of testing, with the time to locate the target decreasing to less than 40 s on day 5 (Fig. 4D, E). The radial maze test measures behavioral patterns by tracking the movement of mice in an 8-armed radial maze; mice with AD cannot evenly search, in contrast to control mice who search all arms of the maze. The behavioral patterns of the mice with AD were irregular, owing to memory deterioration, as expected; thus, the p16^ink4a^ siRNA NP-treated mice had improved memory, as measured by the radial maze test (Fig. 4F). Taken together, all data confirmed that treatment with NPs that reduce expression of p16^ink4a^ delayed the progression of dementia-related learning and memory behaviors.

### P16^ink4a^ knockdown reduces amyloid-β plaque formation and peri-plaque microglia, positively regulates the G1 specific cell cycle

We quantified Aβ levels and amyloid plaque burden in the cortex and hippocampus of mice treated with scrambled siRNA NPs or p16^ink4a^ siRNA NPs. Plaque accumulation was strongly reduced in p16^ink4a^ siRNA NP-injected mice, which resulted in smaller plaque sizes in the cortex and hippocampus (Fig. 4G). Microglial reactivity in the cortex and hippocampus of p16^ink4a^ siRNA NP-injected mice was decreased in peri-plaque regions, which displayed increased Iba1-positive surface areas and numbers of Iba1-positive cells (Fig. 4H). We then used ELISA to quantify the cortex brain levels of Aβ1-42. Compared with scrambled siRNA NPs, p16^ink4a^ siRNA NPs mice showed a two-fold reduction in Aβ1-42 levels per 50 μg cortex (Fig. 4I). In addition, we investigated whether the cell cycle of microglia can be controlled by decreasing the expression of p16^ink4a^ through siRNA capsulated PLGA NPs. P16^ink4a^ specifically inhibits cyclin-dependent kinases 4 and 6 (CDK4, CDK6)-mediated phosphorylation of p-Rb, consequently blocking cell cycle progression [43]. Therefore, we observed an increase in the expression of p-Rb in the group in which the expression of p16^ink4a^ was decreased, which is expected to activate the cell cycle in brain tissue. Furthermore, p16^ink4a^ transmits signals through competitive binding between cdk4/6 and cycline D1, and it can inhibit the activity of CDK4/6 by impairing the binding of the activator cyclin D1 [44]. In our results, decreased expression of p16^ink4a^ caused increased expression of cyclin D1 and decreased expression of cyclin B1 in the cortex of 5XFAD mice (Fig. 4J, 4K). Consequently, downregulating p16^ink4a^ expression through siRNA increases cell division by regulating cell division-related factors.

### Replacing aged microglia to young microglia using p16.^ink4a^ siRNA increases lysosomal activity and reduces ineffective disease- associated microglia (DAM)

**Fig. 5:**
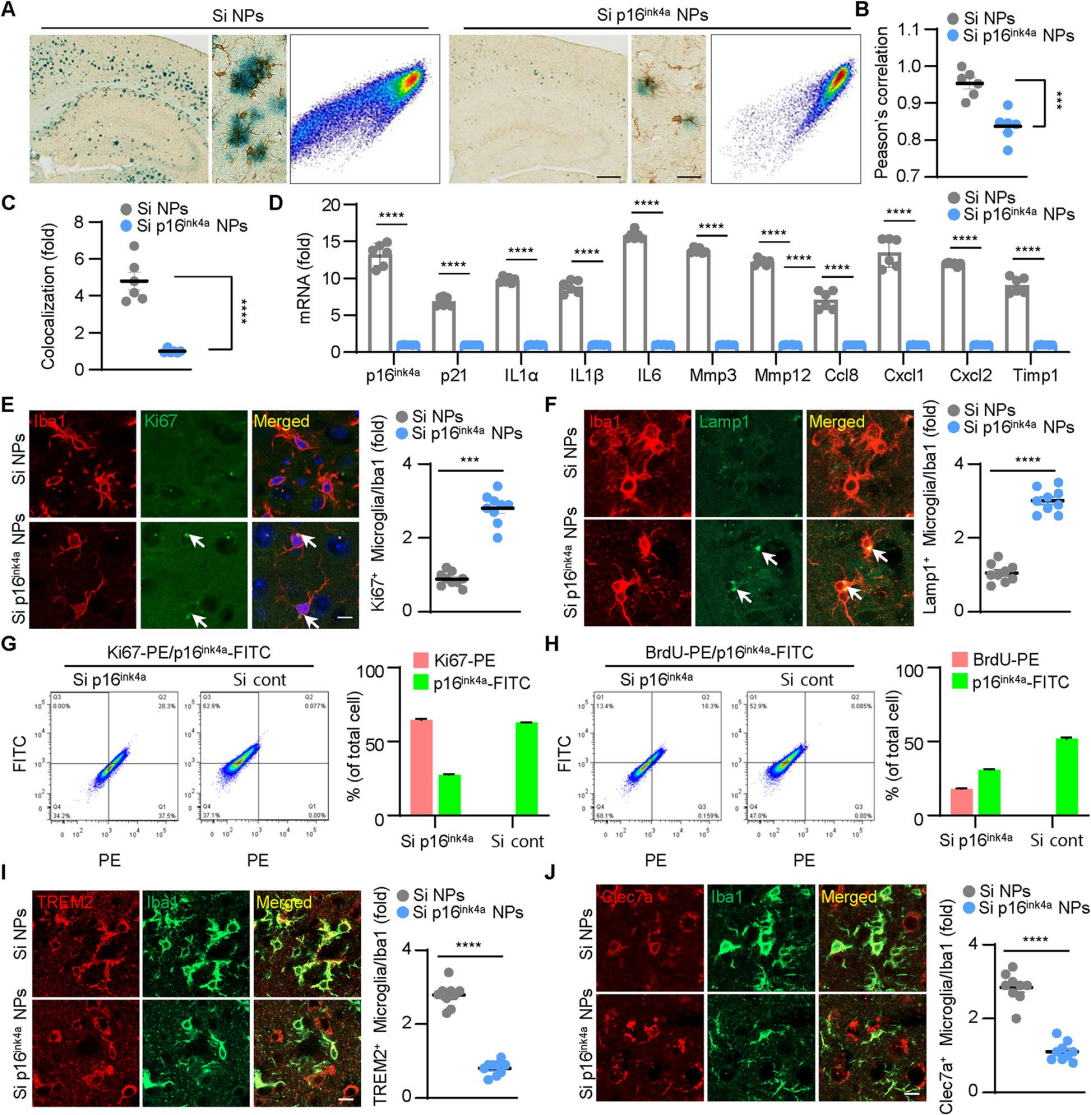
Decreased expression of p16^ink4a^ reduces the number of senescent microglia and initiates new cell divisions, reducing dysfunctional disease-associated microglia (DAM). **A** SA-β-gal activity (blue) and immunostaining with anti-Iba1 antibody (brown). Yellow arrows indicate senescent cells (blue) that merge with Iba1-stained cells (brown). The second images indicate the magnified area. Scale bar: (left) 300 µm, (right) 20 µm. The third images show scatter plot of each staining images. **B** Comparison of Pearson’s correlation coefficient for Iba1 channel and SA-β-gal activity channel in brain tissue. ***p < 0.005, versus scrambled siRNA NPs; unpaired Student’s t test. **C** Graph representing results of colocalization analysis the colocalization plugin from ImageJ program. Results are presented as the mean ± SEM. ****p < 0.001, versus p16^ink4a^ siRNA NPs; unpaired Student’s t test (*n* = 6). **D** The mRNA levels of the cyclin-dependent kinase inhibitors, p21, and a panel of SASP factors were determined by RT-qPCR. ****p < 0.001, versus p16^ink4a^ siRNA NPs; unpaired Student’s t test (*n* = 6). **E** Co-localization of Ki67 (proliferative marker) and Iba1 (microglia marker). White arrows indicate microglia merged with Ki67. Scale bar: 20 µm. ***p < 0.005, versus scrambled siRNA NPs; unpaired Student’s t test. **F** Co-localization of Lamp1 (Lysosomal activity marker) and Iba1 (microglia marker). White arrows indicate microglia merged with Lamp1. Scale bar: 20 µm. ****p < 0.001, versus scrambled siRNA NPs; unpaired Student’s t test. **G** Dot plots from a representative BV2 cells with transfected p16^ink4a^ siRNA and scrambled siRNA are shown. The plot shows the increased expression of ki67 in the p16^ink4a^ siRNA transfected BV2 cell group compared scrambled siRNA transfected cell group. All data were used for each experiment that was repeated three times. **H** Dot plots from a representative BV2 cells with transfected p16^ink4a^ siRNA and scrambled siRNA are shown. The plot shows the increased expression of BrdU in the p16^ink4a^ siRNA transfected BV2 cell group compared scrambled siRNA transfected cell group. 45 h after siRNA transfection, BrdU was treated for 3 h. All data were used for each experiment that was repeated three times. **I** Co-localization of TREM2 (Disease associated microglia marker) and Iba1 (microglia marker). Scale bar: 20 µm. ****p < 0.001, versus scrambled siRNA NPs; unpaired Student’s t test. **J** Co-localization of Clec7a (Disease associated microglia marker) and Iba1 (microglia marker). Scale bar: 20 µm. ****p < 0.001, versus scrambled siRNA NPs; unpaired Student’s t test

To investigate whether microglia around amyloid β plaque was senescent, we performed SA-β-gal staining of 5XFAD mouse brain, followed by co-localization analysis using Scatter J plugin in Image J program. In SA-b-gal staining, surrounding microglia arranged in a circle appear blue, indicating that the microglia around the plaque are aged [45]. And peri-plaque aged microglia also harbored significantly higher senescence-associated β-galactosidase (SA-β-gal) activity, whereas microglia with reduced p16^ink4a^ expression showed significantly lower senescent activity (Fig. 5A). These data show that p16^ink4a^ siRNA NP treatment reduces amyloid plaque deposition, decreases the number of aged microglia, and increases the number of newly proliferated microglia. Pearson’s coefficient of these co-staining images showed that Iba1 had the strongest co-localization with SA- β-gal. The X-axis represents the brightness of the Iba1 channel and y-axis represents the brightness of the SA-β-gal channel (Fig. 5B). Theoretically, if the fluorescence distributions of the two channels are similar, that is, if the colocalization is good at the same spot, the scatter plot will be concentrated along the diagonal. And as we expected, microglia are significantly weaker in co-localization with SA-β-gal in p16^ink4a^ siRNA NP-injected mice. (Fig. 5C).

Next, we confirmed the expression of senescence markers in the brain tissue of mice injected with p16^ink4a^ siRNA NPs compared to scrambled siRNA NPs. P16^ink4a^ siRNA worked well, reducing mRNA expression. To determine the expression of senescence markers, we compiled a list of 10 well-established SASP factors, including p21 (one of the cellular senescence markers), IL1α, IL1β, IL6, Mmp3, Mmp12, Ccl8, Cxcl1, Cxcl2, and Timp1 [46, 47]. We observed significant differences among each group of mice. Upon comparing the expression levels between mice injected with p16^ink4a^ siRNA and those injected with scrambled siRNA NPs, we found that all aging-related factors were increased in the scrambled siRNA NPs group (Fig. 5D).

As further confirmation, we quantified the proliferation marker Ki67 with Iba1 to determine whether p16^ink4a^ knockdown with NPs contributed to an increase in the number of younger microglia than aged microglia. Interestingly, staining of mouse brain sections revealed that injection with p16^ink4a^ siRNA NPs led to a significantly higher number of proliferating microglia (Iba1 + /Ki67 +) when measured against the total number of microglia (Iba1 +) in the cortex (Fig. 5E). In addition, to check the lysosomal function of microglia in p16ink4a siRNA NP-injected mice, Lamp1 (a marker of lysosomes) was immunostained. As expected, microglia (Iba1/Lamp1 +) increased 3.4-fold compared to the control group (Fig. 5F). These observations, along with previous evidence that senescent microglia are reduced and rejuvenated through down-expression of p16^ink4a^, indicate greater cell cycle re-entry. Additional experiments were performed to confirm cell division through FACS analysis, and the number of ki67-positive cells increased by 64.4% in the microglial cell line BV2 treated with p16^ink4a^ siRNA (Fig. 5G). And then, we determine whether p16^ink4a^ siRNA transfected BV2 cell affected the proliferative capacity, cells were cultured with Bromodeoyuridine (BrdU) for 3 h before harvest. Cells treated with p16^ink4a^ siRNA detected 17.8% higher BrdU than cells treated with control siRNA, indicating a higher proliferation rate. These data suggest that reduced expression of p16^ink4a^ promotes the proliferative capacity of senescent microglia (Fig. 5H).

Recent reports suggest that microglia, or at least a subset of them, have a senescence-activated phenotype. Further confirmation of these findings over the years has corroborated the idea that aged microglia have a priming phenotype that tends to respond more strongly to stimuli, rather than a more inflammatory state in normal condition [48]. A new microglial cell type associated with neurodegenerative diseases has been defined as Disease associated microglia (DAM). In AD mice, TREM2 is upregulated in DAM observed in AD models. Also, Clec7a (also known as Dectin-1) was upregulated in a TREM2- and APOE-dependent manner. TREM2 and Clec7a-positive microglia were found adjacent to Aβ plaques and are known to be overexpressed in AD [49]. Indeed, in our AD model, the expression of TREM2(Fig. 5I) and Clec7a (Fig. 5J) was high in microglia around the amyloid plaque. Interestingly, we found a decrease in dysfunctional DAM in brain tissue with reduced expression of p16^ink4a^. Combined, these data suggest that low expression of p16^ink4a^ contributes to reducing old microglia and increasing the fraction of young microglia. Consequently, it contributes to the overall improvement of phagocytosis of Aβ peptides during onset of Alzheimer's disease.

### Reduction in p16^ink4a^ expression in microglial BV2 cells enhances amyloid-β phagocytosis by stimulating cell cycle re-entry

**Fig. 6:**
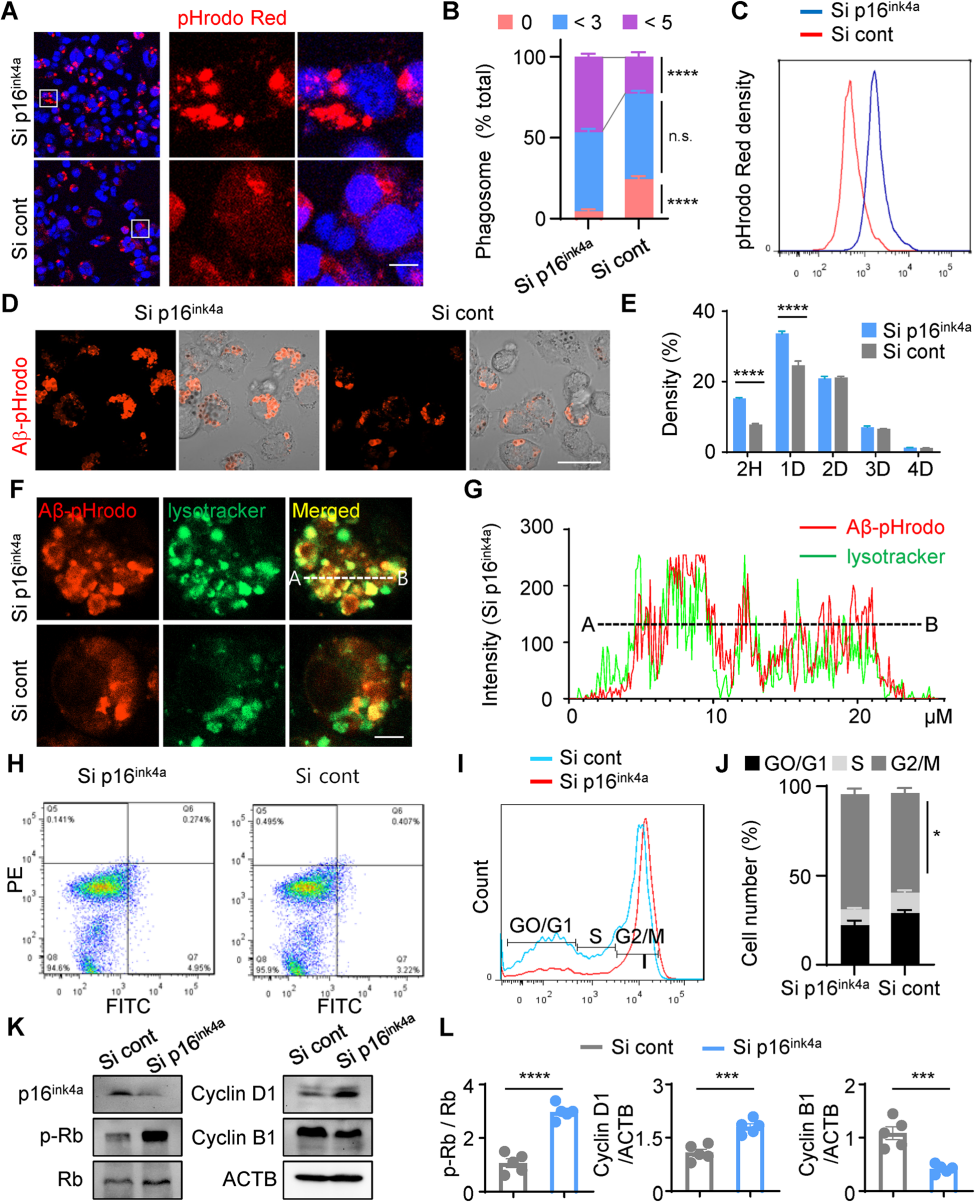
Downregulation of p16^ink4a^ improves microglial BV2 cell proliferation and efficiently increases cellular phagocytosis and amyloid-β lysis. **A** Confocal images of BV2 cells transfected with p16^ink4a^ siRNA or scrambled siRNA and, 48 h later, treated with pHrodo bioparticle conjugates for 3 h. Scale bar: 20 µm. **B** The percentage of the number of phagosomes in each BV2 cell, separated into groups of 0, < 3, and < 5 phagosomes per cell. Data were analyzed by 2-way ANOVA followed by the Tukey test for multiple comparisons. ****p < 0.001, versus scrambled siRNA. n.s.: not significant. **C** FACS analysis of pHrodo-treated BV2 cells. **D** BV2 cells transfected with siRNA were treated with Aβ-pHrodo for 1 h. Scale bar: 20 µm. **E** BV2 cells were treated with Aβ-pHrodo for 2 h, harvested, and analyzed by FACS immediately (2 h after treatment) and on days 1, 2, 3, and 4 after treatment. Data were analyzed by 2-way ANOVA followed by the Tukey test for multiple comparisons. ****p < 0.001, versus scrambled siRNA. D: day, H: hour. **F** Merged representative images of BV2 cells treated with Aβ-pHrodo and LysoTracker. Scale bar: 5 µm. **G** Analysis of the fluorescence intensity along the white line from A to B from the upper merged image in (**F**). **H** Representative FACS data of BV2 cells double-stained with annexin V and propidium iodide. The 4 populations are non-apoptotic dead cells (Q5), late-apoptotic cells (Q6), viable cells (Q8), and early-apoptotic cells (Q7). **I** Representative FACS data of the cell cycle phases of BV2 cells transfected with scrambled siRNA or p16^ink4a^ siRNA. **J** The percentages of cells in G0/G1, S, and G2/M phases for p16^ink4a^ siRNA-transfected BV2 cells compared with scrambled siRNA-transfected control BV2 cells. This experiment was repeated 3 times, and similar results were obtained each time. Data were analyzed by 2-way ANOVA followed by the Bonferroni test for multiple comparisons. *p < 0.05, versus scrambled siRNA. **K** Western blot of p16^ink4a^, p-Rb, Rb, cyclin D1, and cyclin B1 expression in BV2 cells. β-Actin (ACTB) was used as a protein loading control. **L** Quantification of the protein levels in (**K**) after siRNA transfection in BV2 cells. ***p < 0.005 and ****p < 0.001, versus scrambled siRNA; unpaired Student’s t test. Cont: control, Si: siRNA

We investigated whether the decrease in aged microglia and the increase in newly proliferated microglia in p16^ink4a^ siRNA NP-treated mice reflects a conversion of aged microglia to young microglia that have enhanced phagocytic ability to remove plaque load. It is well known that the phagocytic function of microglia is lost in patients with AD, despite microglial activation [50]. To determine whether it is possible to increase microglial phagocytosis by reducing p16^ink4a^ expression, we transfected the microglial BV2 cell line with p16^ink4a^ siRNA NPs or scrambled siRNA NPs. After 48 h, we treated the cells with pH-sensitive red fluorescent pHrodo bioparticle conjugates to observe changes in phagocytosis (Fig. 6A). The proportion of the cells with more than three pHrodo-fluorescent phagosomes was higher in BV2 cell treated with p16^ink4a^ siRNA NPs compared to those of siRNA NPs (Fig. 6B). The number of phagosomes contained in the cells increased remarkably after reduction of p16^ink4a^ expression, and more than 31.79% of these cells had increased phagocytic activity. In addition, fluorescence-activated cell sorting (FACS) analysis confirmed that cells with reduced p16^ink4a^ expression had increased red fluorescence (Fig. 6C). Then, we conjugated Aβ (1–42) to pHrodo bioparticle conjugates to reveal how microglial phagocytosis directly affects Aβ clearance. BV2 cells transfected with scrambled siRNA or p16^ink4a^ siRNA were treated with Aβ (1–42)-conjugated pHrodo (Aβ-pHrodo) for 1 h. As expected, BV2 cells with reduced p16^ink4a^ expression and treated with Aβ-pHrodo contained more pHrodo-fluorescent vesicles (Fig. 6D). We also measured red fluorescence intensity in BV2 cells after treatment with Aβ-pHrodo for 2 h, 1 day, 2 days, 3 days, and 4 days by FACS analysis (Fig. 6E). Initially, Aβ-pHrodo phagocytic uptake was increased when p16^ink4a^ expression was reduced; however, after 2 days, the Aβ-pHrodo phagocytic uptake was similar to that of the control group. This suggests that Aβ was taken up at significantly increased levels in BV2 cells with reduced p16^ink4a^ expression. Next, we determined whether phagocytosed Aβ was lysed in BV2 cells with reduced p16^ink4a^ expression and found co-localization of Aβ-pHrodo and a lysosome-sensing LysoTracker dye (Fig. 6F). We measured the fluorescence intensity across confocal images to show that the locations of Aβ-pHrodo and LysoTracker intensity profiles strongly correlated in cells treated with p16^ink4a^ siRNA NPs (Fig. 6G).

P16^ink4a^ is a cell cycle regulator and promotes cell cycle arrest from the G1 phase to the S phase by inhibiting binding of CDK4/6 with cyclin D1 [51]. Annexin V and propidium iodide staining were performed to confirm whether dysregulation of the cell cycle caused by treatment with p16^ink4a^ siRNA NPs affected cell death. Reduction in p16^ink4a^ expression did not affect necrosis or apoptosis (Fig. 6H). FACS analysis verified that the cell cycle was altered by a reduction in p16^ink4a^ expression, as the number of cells shifted to the G2/M phase increased from 55.75% to 64.45% (Fig. 6I, J). Although there was no significant quantitative change in the number of cells in S phase between the 2 groups, we expected the number of cells in G0/G1 to decrease and cell cycle re-entry to increase after reduction in p16^ink4a^ expression levels. As p16^ink4a^ functions as a CDK inhibitor, cell cycle progression from G1 to S phase requires the activation of CDK4 and CDK6, which inactivate the retinoblastoma (Rb) tumor-suppressor protein by phosphorylation [52]. As we expected, p16^ink4a^ siRNA increased Rb phosphorylation, which promotes S phase entry, and increased the expression of cyclin D1 (Fig. 6K, L). Reduced expression of p16^ink4a^ induces its assembly with cyclin D1 and is thus activated, leading to cell cycle progression in G1. Taken together, these data confirm that a reduction in p16^ink4a^ expression increases the phagocytic and lytic functions of microglia by stimulating cell cycle re-entry in microglia cell line.

## Discussion

Cellular senescence is an irreversible arrest of cell growth [53, 54]. Cellular senescence can be triggered by several factors including aging, DNA damage, oncogene activation, and oxidative stress [55]. Although the molecular mechanisms of senescence involve p16^ink4a^, the *p53* tumor-suppressor gene, and telomere shortening [56], this study focused on the mechanisms of p16^ink4a^ control. Regulation of p16^ink4a^ expression is complex and involves epigenetic control and multiple transcription factors. P16^ink4a^ mediates senescence and aging in microglia by G1 cell cycle arrest [57, 58]^.^ by preventing the association of CDK4 and CDK6 to D-type cyclins. This process is regulated through the Rb pathway; Rb is maintained in a hypophosphorylated state, resulting in the inhibition of transcription factor E2F1. The expression of p16^ink4a^ is increased in microglia from patients with AD, as quantified by single-cell transcriptomic analysis [59]. However, it is unclear whether increased p16^ink4a^ expression causes a decline in microglial function during aging *in vivo*.

Although the importance of microglia in AD pathology has recently increased, there is a lack of studies on the recovery of microglial function and therapeutic approaches for AD by direct regulation of microglial gene expression. This is because researchers have tested various AAV serotypes and lentiviruses for their ability to transduce microglia, and the level of transduction is generally low [16]. siRNA-mediated gene silencing is an effective and powerful tool, but its therapeutic effects are limited because of suboptimal delivery to the target site and because siRNAs are easily degraded in vivo. Here, we used PLGA NPs to deliver siRNA to the target site and to improve the pharmacokinetics and stability of the siRNA. PLGA NPs have negatively charged surfaces and a neutral pH, and after endocytic internalization, biodegradable PLGA NPs undergo surface charge reversal caused by a pH change from anionic (the physiological and alkaline pH) to cationic (the endosomal and lysosomal acidic pH). This facilitates the interaction of NPs with the endolysosomal membrane and allows their escape into the cytoplasm [19, 60, 61]. Our group previously generated several types of gene regulators encapsulated in PLGA NPs, which successfully targeted microglia. The unique characteristic of microglia as macrophages allows them to take up NPs by phagocytosis, which other nervous system cells cannot do. NPs promote the delivery of higher concentrations of encapsulated drugs or gene mediators to microglia [20–24]. Flow cytometry analysis has shown that drug-loaded NPs can facilitate microglial phenotypes, recovery of the M1/M2 macrophage ratio, and anti-inflammatory activity [20]. Here, we present evidence to implicate senescent microglia in the progression of neurodegenerative diseases such as AD by increasing our knowledge of the mechanistic contribution of these cells that may actively drive neurodegeneration and by investigating how these cells or their effects may be targeted therapeutically. We focused on the aging of microglia as the most important factor for AD progression. Chronic microglial reactivity, commonly observed in AD [62], stimulates an acute immune response against misfolded proteins, such as Aβ, that may actively drive neuronal death through excessive production of neurotoxic factors [63]. Indeed, there is a lack of evidence as to the functional differences between young and aged microglia. According to a recent report, aged microglia could clear amyloid plaques under the influence of young microglia, which suggests that aged microglia could have a constructive function in the brain. The authors improved the function of young microglia, which increased amyloid plaque clearance and reduced Aβ plaque load [64]. However, this report did not reliably explain the dysfunction of phagocytosis in aged microglia.

Another hypothesis is that stem cells may lose their regenerative capacity as they age, resulting in a lack of healthy microglia [65]. The resultant increase in aged and dysfunctional microglia without protective immunity would reduce their response to protective immunity in a state such as AD. Microglial proliferation increases as AD progresses, which is reflected in microglial accumulation as markers around plaques significantly upregulated. However, there is insufficient evidence to explain the proliferation potential of microglia and its consequences in AD. Therefore, if the phagocytic efficiency of aged microglia is the cause of AD, then improvement of the microglial scavenging mechanism could be a treatment for AD.

Senescent microglia have also been identified as contributing to the pathogenesis of neurodegenerative diseases [66]. And the morphological definition of senescent microglia is distinctly different from that of activated microglia [67]. Morphologically speaking, dystrophic microglia are also increased in several neurodegenerative diseases. And the relationship between aging and 'activated' microglia in the context of disease is unclear [13]. Because this phenotype has been described as having features of both M1 and M2 microglia, further weakening the concept of activated microglia dichotomy. This is particularly challenging because Senescence associated secretory phenotype (SASP), a key function of senescent cells, typically contains pro-inflammatory molecules associated with 'activated' microglia, such as TNFα, IL-1β and IL-6 [68]. It is also unclear whether aged microglia upregulate markers associated with 'activated' microglia or downregulate microglia homeostasis genes. However, what is certain is that many studies are being conducted on the difference between recently activated microglia and disease-associated microglia (DAM) [59]. It starts by reporting changes in the expression of markers such as TREM2 and clec7a [69]. Given the dysfunctional phagocytosis in aged microglia, old microglia that did not phagocytose Aβ42 had altered expression of these genes.

In this study, we revealed that enhancement of microglial phagocytosis is protective in AD by promoting Aβ clearance and thus delaying disease progression. However, it is uncertain whether microglia degrade amyloid aggregates. If amyloid aggregates are not degraded after phagocytosis, then Aβ removal is difficult; accumulated Aβ proteins may be toxic and pro-inflammatory to the microglia. Indeed, microglia from patients with AD that carry TREM2 risk variants and TREM2-deficient mice with AD-like pathology have many autophagic vesicles, as do TREM2-deficient macrophages under growth factor limitation or endoplasmic reticulum stress [70]. This suggests that Aβ is engulfed by microglia but not lysed intracellularly. From a therapeutic perspective, Aβ removal involves either decomposition by phagocytosis and lysis in microglia or excretion out of the brain. Therefore, engulfment of Aβ into microglia without subsequent lysis is an example of phagocytic dysfunction. Microglial phagocytic dysfunction may be caused by reduced phagocytosis or by excessive Aβ uptake beyond their phagocytic capacity. Ultimately, products that accumulate in microglia due to aging or other causes can cause dysfunction and activation of microglia, resulting in a chronic inflammatory state. In therapeutic terms, anti-inflammatory drugs may reduce this response to aging and AD-related neuroinflammation.

Microglia have differing effects on plaque dynamics depending on the overall plaque burden. It has been argued that microglia, or products they release, are detrimental during certain stages of AD progression [71]. In contrast, in AD and other neurodegenerative diseases, microglia secrete cytokines, phagocytose Aβ plaques, and physically restrain the expansion of plaques to protect nearby healthy neurites [72]. A recent study using the 5XFAD mouse model with PLX5622, a specific colony-stimulating factor 1 receptor inhibitor that allows for the sustained and specific elimination of microglia [71], was conducted to determine whether microglial depletion influences AD pathology [72]. Here, we present evidence that microglia are required to remove the majority of dense plaques. Plaque morphologies revert to control levels upon microglial repopulation. Overall, in regions where microglia have repopulated to control levels, microglia encourage the remodeling of plaque types toward compact-like structures and limit neuritic dystrophy. This means that repopulated microglia inhibit and eliminate plaque formation during AD progression. Our study also suggests that repopulated microglia have active phagocytic activity and are more efficient at Aβ clearance than their younger counterparts.

## Conclusion

In summary, we provide a proof-of-concept study toward the use of p16^ink4a^ siRNA-loaded PLGA NPs to enhance Aβ phagocytosis and clearance by causing a shift to a younger microglial phenotype by stimulating cell cycle re-entry. We also add to the substantial body of work that has demonstrated the nanomedicinal application of p16^ink4a^ genetic knockdown that improves amyloid pathology and rescues cognitive function in mouse models of AD. First, we found microglia are activated near and co-localize with Aβ plaques in 5XFAD mice. Next, we found that p16^ink4a^, a representative factor of aging and prevention of cell cycle progression from G1 to S, was increased in a list of DEGs in Aβ42-positive microglia from old mice compared with young mice. High expression of p16^ink4a^ in microglia was confirmed around amyloid plaques in patients with AD and in 5XFAD mice. As young microglia have increased amyloid plaque clearance and reduced Aβ plaque load [64], we hypothesized that rejuvenation of the microglial cell-division cycle could reverse the phagocytic dysfunction of aged microglia. To shift aged microglia to a younger phenotype, we used p16^ink4a^ siRNA-loaded PLGA NPs, which successfully targeted microglia, activated microglia, and prolonged gene-regulatory effects. P16^ink4a^ knockdown in microglia using p16^ink4a^ siRNA-loaded NPs reversed spatial memory and learning deficits and reduced Aβ plaque formation and the number of peri-plaque aged microglia in 5XFAD mice. A reduction in p16^ink4a^ expression increased the phagocytic and lytic functions of microglia by stimulating cell cycle re-entry. In addition, cell cycle re-entry was induced, resulting in increased cell proliferation. Importantly, our results implicate a protective role for p16^ink4a^ siRNA-loaded NPs in aging-related microglial phagocytic dysfunction and reduced Aβ clearance. p16^ink4a^ siRNA-loaded NPs could be a potentially therapeutic technology to reduce Aβ accumulation-associated degeneration in patients with AD and other microglial dysfunction-related disorders.

### Supplementary Information

**Additional file 1: Supplementary Figure 1.** 5XFAD mice have increased amyloid-b plaque formation over time, which induces glial cell activation near amyloid-b plaques. (A) Expression of Ab in 5XFAD mouse brains at 2, 3, 4, and 8 months of age. Ab was labeled using a rabbit monoclonal antibody.Representative images of hippocampal and cortical parasagittal serial sections are shown. Scale bar: 200 µm. (B) and (C) Brain tissue from 8-month-old 5XFAD mice was used for immunostaining with anti-Iba1 (microglial marker), anti-GFAP (astrocyte marker), and anti-NeuN (neuronal marker) antibodies. Representative images of hippocampal (B) and cortical (C) parasagittal serial sections are shown. Scale bar: 200 µm. (D) Western blot of the protein levels of GFAP, Ab, Iba1, NeuN, and b-actin (ACTB) in the cortex of 5XFAD mice at 8 months of age and their age-matched littermate wild-type (WT) controls. (E) Quantification of Western blot data of GFAP, Ab, IbaI, and NeuN protein expression in relation to ACTB expression. *p < 0.05 and ****p < 0.001, versus WT (unpaired Student’s t test; n = 4 for each group).

**Additional file 2: Supplementary Figure 2. **The number of peripheral microglia correlates with the size of amyloid-β plaques. (A) Co-localization of Aβ and Iba1(microglia marker) in hippocampus of 5XFAD mouse. The white boxes (a, b) show the areas magnified in the lower left panels. Scale bar: 50 µm (upper), 20 µm (lower). (B) Quantification of Ab plaque size and Iba1-positive cell numbers. Each black dot indicates 1 Ab plaque (n = 210). Pearson correlation analysis showed that Ab plaque size correlated with Iba1-positive cell numbers (r = 0.9964, *p <* 0.0001, n = 210).

**Additional file 3: Supplementary Figure 3. **Differential expression of *CDKN2A* in relation to human brain regions and ages from Genotype-Tissue Expression (GTEx) data. Comparison of *CDKN2A* expression in 12 brain regions separated by subject age.All groups were compared with the 20- to 29-year age group using the Kruskal–Wallis test and the Dunn multiple comparisons test. Anterior cingulate cortex, 30-39 years, *p*= 0.05; cortex, 60-69 years, *p* = 0.0028; cortex, 70-79 years, *p* = 0.0075; frontal cortex Brodmann area 9, 60-69 years, *p* = 0.034; hippocampus, 60-69 years, *p* = 0.0148; hippocampus, 70-79 years, *p*= 0.0157. Age groups on the graphs without p-value asterisks were non-significant, *p* > 0.05.

**Additional file 4: Supplementary Figure 4.**Up-regulation of p16^ink4a^ expression of microglia in postmortem brains of patients with Alzheimer’s disease (A, B) Details of human post-mortem cortex tissues from the Victorian Brain Bank Network. PMI, post-mortem interval. (C) Western blot of the protein expression levels of p16^ink4a^, Ab, Iba1, and β-actin (ACTB) in the human cortex of age-matched controls and patients with AD. (D) Quantification of protein expression relative to ACTB from (C). **p < 0.005, patients with AD versus healthy controls (unpaired Student’s t test; n = 5 for each group). Cont: control.

**Additional file 5: Supplementary Figure 5.** Poly(D,L-lactic-co-glycolic acid) nanoparticles encapsulated with p16^ink4a^ siRNA attenuated the expression of p16^ink4a^. (A) mRNA expression of p16^ink4a^ was quantified by qPCR in the cortex of 4-month-old 5XFAD mice injected intrathecally into the cisterna magna with scrambled siRNA NPs or p16^ink4a^ siRNA NPs once per week for 8 weeks. ****p < 0.001, versus mice treated with scrambled siRNA NPs; unpaired Student’s t test. (B) Western blot of p16^ink4a^ expression in brain tissue of 4-month-old 5XFAD mice injected with scrambled siRNA NPs or p16^ink4a^ siRNA NPs once per week for 8 weeks. (C) Quantification of (B). ACTB: β-actin. ****p< 0.001, versus mice treated with scrambled siRNA NPs; unpaired Student’s t test. (D) Immunostaining with anti-p16^ink4a^ antibody of cortex brain sections from 8-month-old 5XFAD mice. Scale bar: 100 µm. (E) Quantification of p16^ink4a^ expression in (C). ****p < 0.001, versus mice treated with scrambled siRNA NPs; unpaired Student’s t test. Si: siRNA.

**Additional file 6: Supplementary Figure 6. **Raw fluorescence images for IMARIS figures.

**Additional file 7: Supplementary Figure 7.** Raw data for western blot.

**Additional file 8: Supplementary Table 1. **Statistical analyses.

**Additional file 9: Supplementary Table 2. **Top 5 gene ontology terms involved with the 418 increased and 375 decreased differentially expressed genes in amyloid-β42-positive microglia from old mice compared with young mice.

## Data Availability

All data generated or analyzed during this study are included in this published article (and its supplementary information files).

## Abbreviations

AD: Alzheimer’s disease
PLGA: Poly (D,L-lactic-*co*-glycolic acid)
Aβ: Amyloid-β
CDK: Cyclin-dependent kinase
Cdkn2a, p16^ink4a^: Cyclin-dependent kinase inhibitor 2A
NPs: Nanoparticles
AAVs: Adeno-associated viruses
siRNA: Small interfering RNA
DEGs: Differentially expressed genes
GTEx: Genotype-Tissue Expression
Aβ-pHrodo: Aβ (1–42)-conjugated pHrodo
Rb: Retinoblastoma
