# Supplementary figures and images for: Rejuvenating aged microglia by p16ink4a-siRNA-loaded nanoparticles increases amyloid-β clearance in animal models of Alzheimer’s disease
Source: Mol Neurodegener. 2024 Mar 16;19:25. doi: 10.1186/s13024-024-00715-x (PMC10943801; doi:10.1186/s13024-024-00715-x)

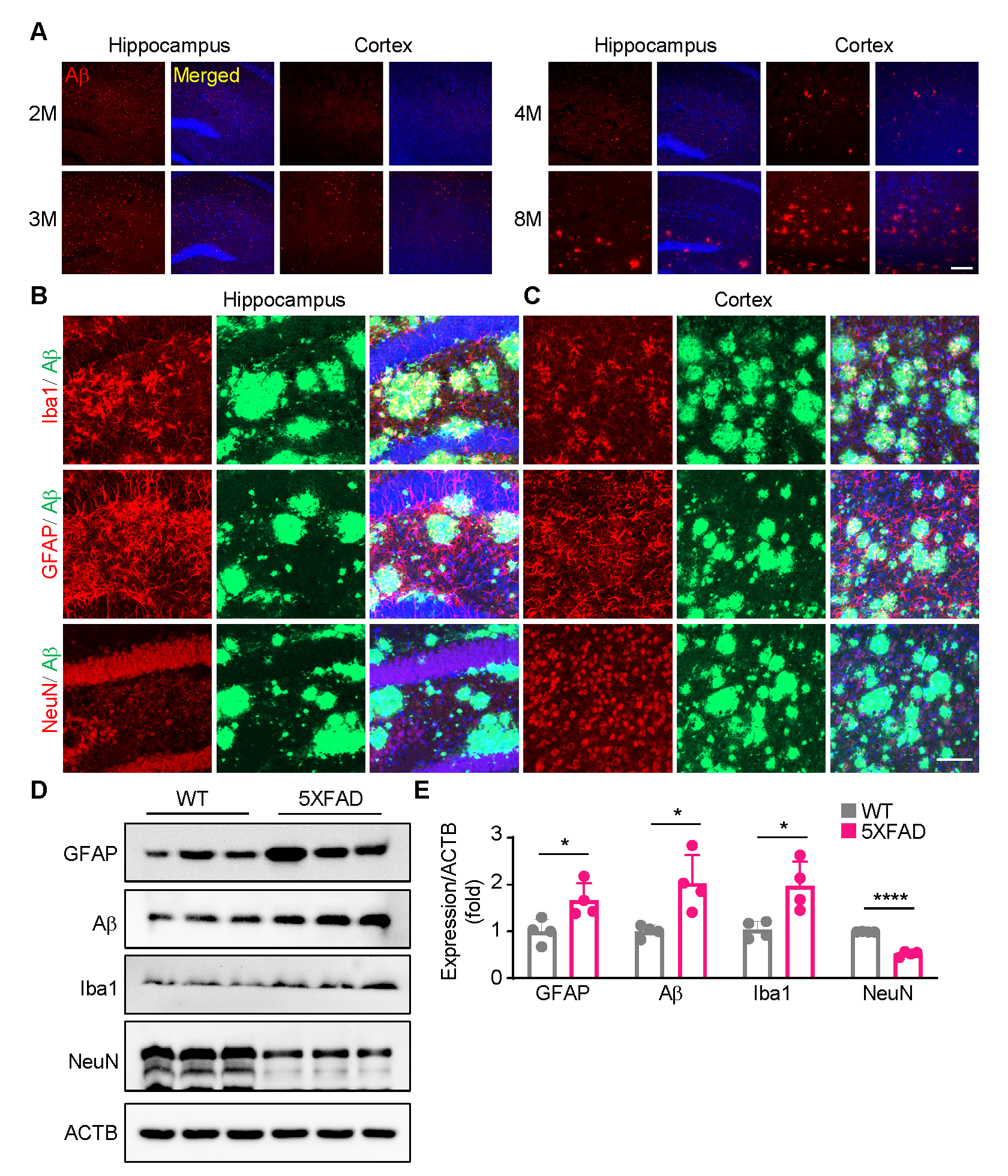

Supplement: Supplementary file 1 — Additional file 1: Supplementary Figure 1. 5XFAD mice have increased amyloid-b plaque formation over time, which induces glial cell activation near amyloid-b plaques. (A) Expression of Ab in 5XFAD mouse brains at 2, 3, 4, and 8 months of age. Ab was labeled using a rabbit monoclonal antibody.Representative images of hippocampal and cortical parasagittal serial sections are shown. Scale bar: 200 µm. (B) and (C) Brain tissue from 8-month-old 5XFAD mice was used for immunostaining with anti-Iba1 (microglial marker), anti-GFAP (astrocyte marker), and anti-NeuN (neuronal marker) antibodies. Representative images of hippocampal (B) and cortical (C) parasagittal serial sections are shown. Scale bar: 200 µm. (D) Western blot of the protein levels of GFAP, Ab, Iba1, NeuN, and b-actin (ACTB) in the cortex of 5XFAD mice at 8 months of age and their age-matched littermate wild-type (WT) controls. (E) Quantification of Western blot data of GFAP, Ab, IbaI, and NeuN protein expression in relation to ACTB expression. *p < 0.05 and ****p < 0.001, versus WT (unpaired Student’s t test; n = 4 for each group). [file 13024_2024_715_MOESM1_ESM.tif]

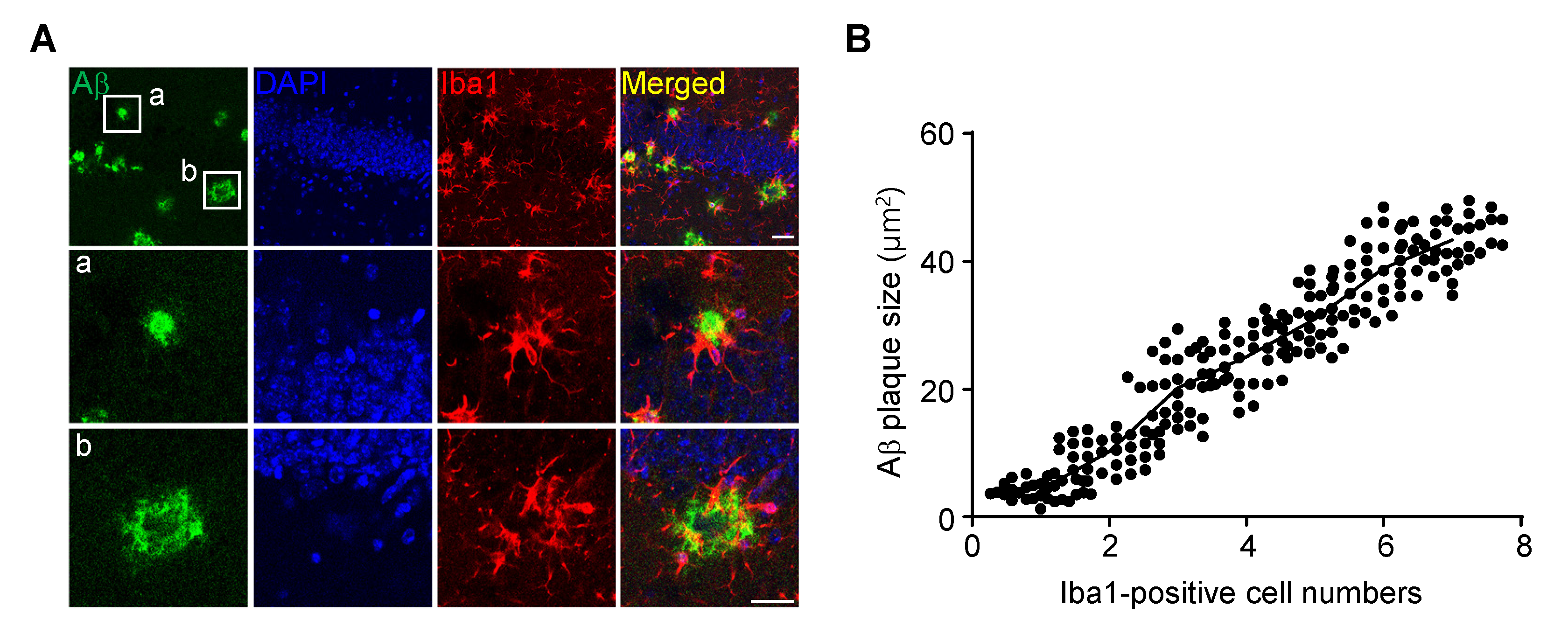

Supplement: Supplementary file 2 — Additional file 2: Supplementary Figure 2. The number of peripheral microglia correlates with the size of amyloid-β plaques. (A) Co-localization of Aβ and Iba1(microglia marker) in hippocampus of 5XFAD mouse. The white boxes (a, b) show the areas magnified in the lower left panels. Scale bar: 50 µm (upper), 20 µm (lower). (B) Quantification of Ab plaque size and Iba1-positive cell numbers. Each black dot indicates 1 Ab plaque (n = 210). Pearson correlation analysis showed that Ab plaque size correlated with Iba1-positive cell numbers (r = 0.9964, p < 0.0001, n = 210). [file 13024_2024_715_MOESM2_ESM.tif]

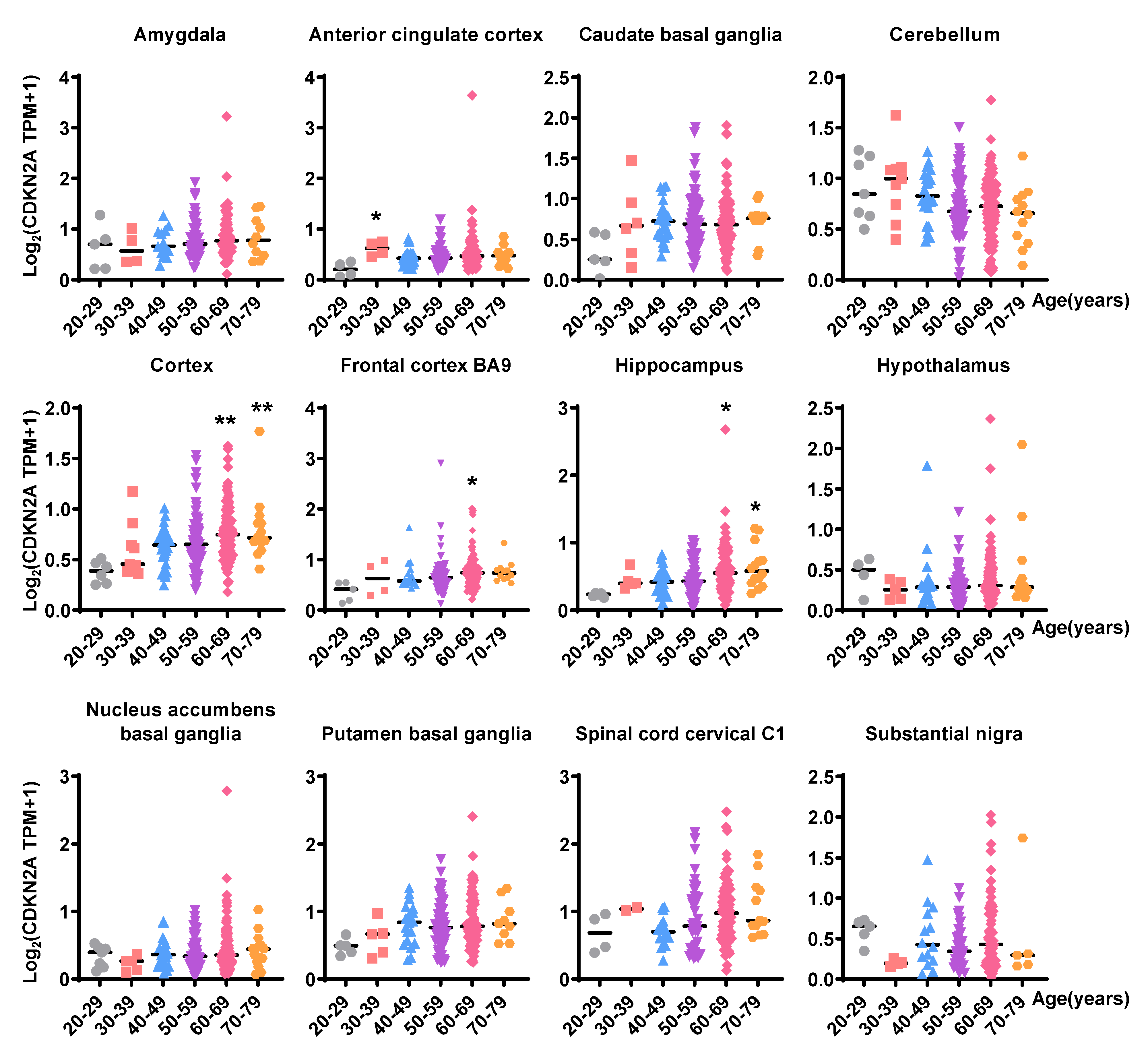

Supplement: Supplementary file 3 — Additional file 3: Supplementary Figure 3. Differential expression of CDKN2A in relation to human brain regions and ages from Genotype-Tissue Expression (GTEx) data. Comparison of CDKN2A expression in 12 brain regions separated by subject age.All groups were compared with the 20- to 29-year age group using the Kruskal–Wallis test and the Dunn multiple comparisons test. Anterior cingulate cortex, 30-39 years, p= 0.05; cortex, 60-69 years, p = 0.0028; cortex, 70-79 years, p = 0.0075; frontal cortex Brodmann area 9, 60-69 years, p = 0.034; hippocampus, 60-69 years, p = 0.0148; hippocampus, 70-79 years, p= 0.0157. Age groups on the graphs without p-value asterisks were non-significant, p > 0.05. [file 13024_2024_715_MOESM3_ESM.tif]

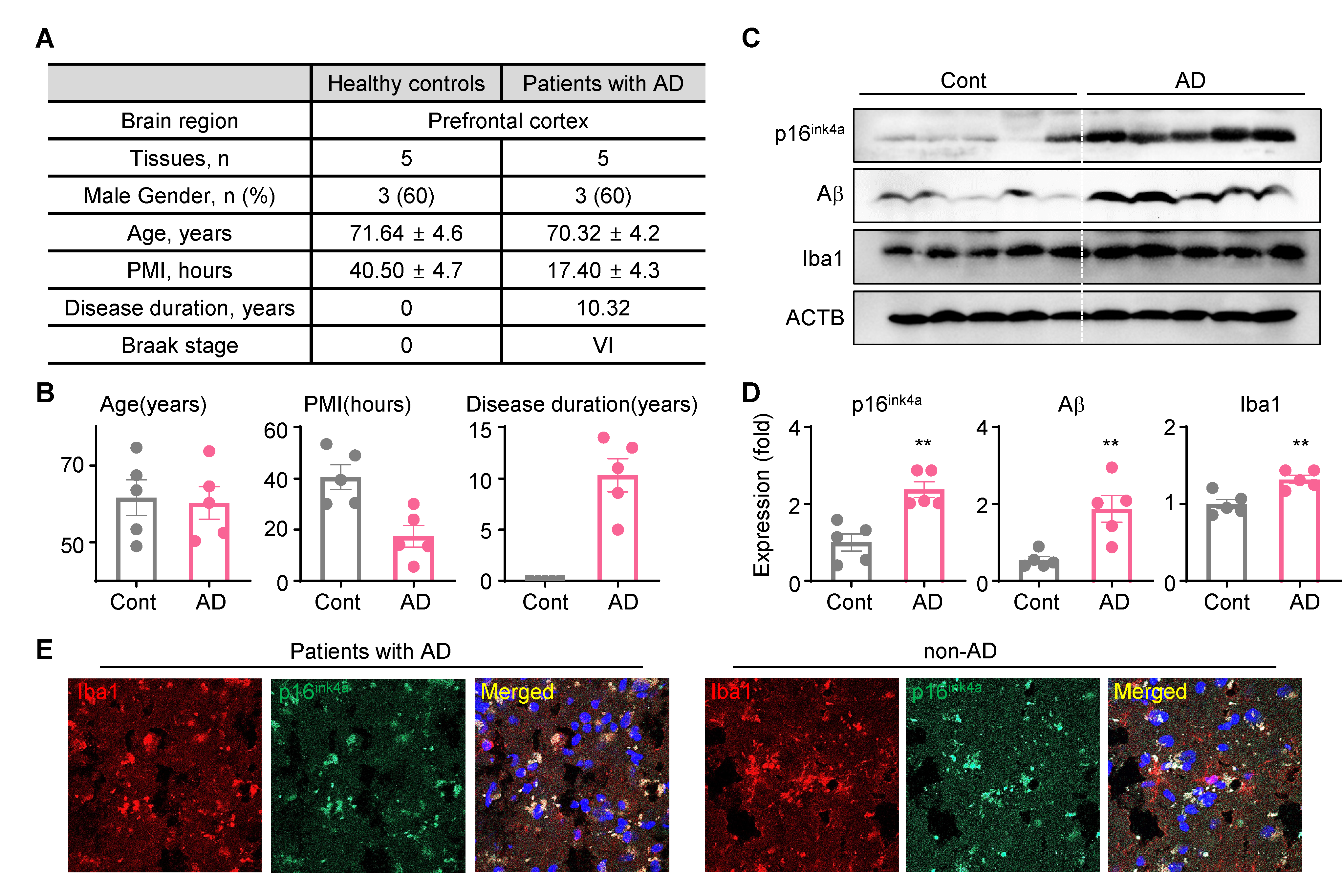

Supplement: Supplementary file 4 — Additional file 4: Supplementary Figure 4.Up-regulation of p16ink4a expression of microglia in postmortem brains of patients with Alzheimer’s disease (A, B) Details of human post-mortem cortex tissues from the Victorian Brain Bank Network. PMI, post-mortem interval. (C) Western blot of the protein expression levels of p16ink4a, Ab, Iba1, and β-actin (ACTB) in the human cortex of age-matched controls and patients with AD. (D) Quantification of protein expression relative to ACTB from (C). **p < 0.005, patients with AD versus healthy controls (unpaired Student’s t test; n = 5 for each group). Cont: control. [file 13024_2024_715_MOESM4_ESM.tif]

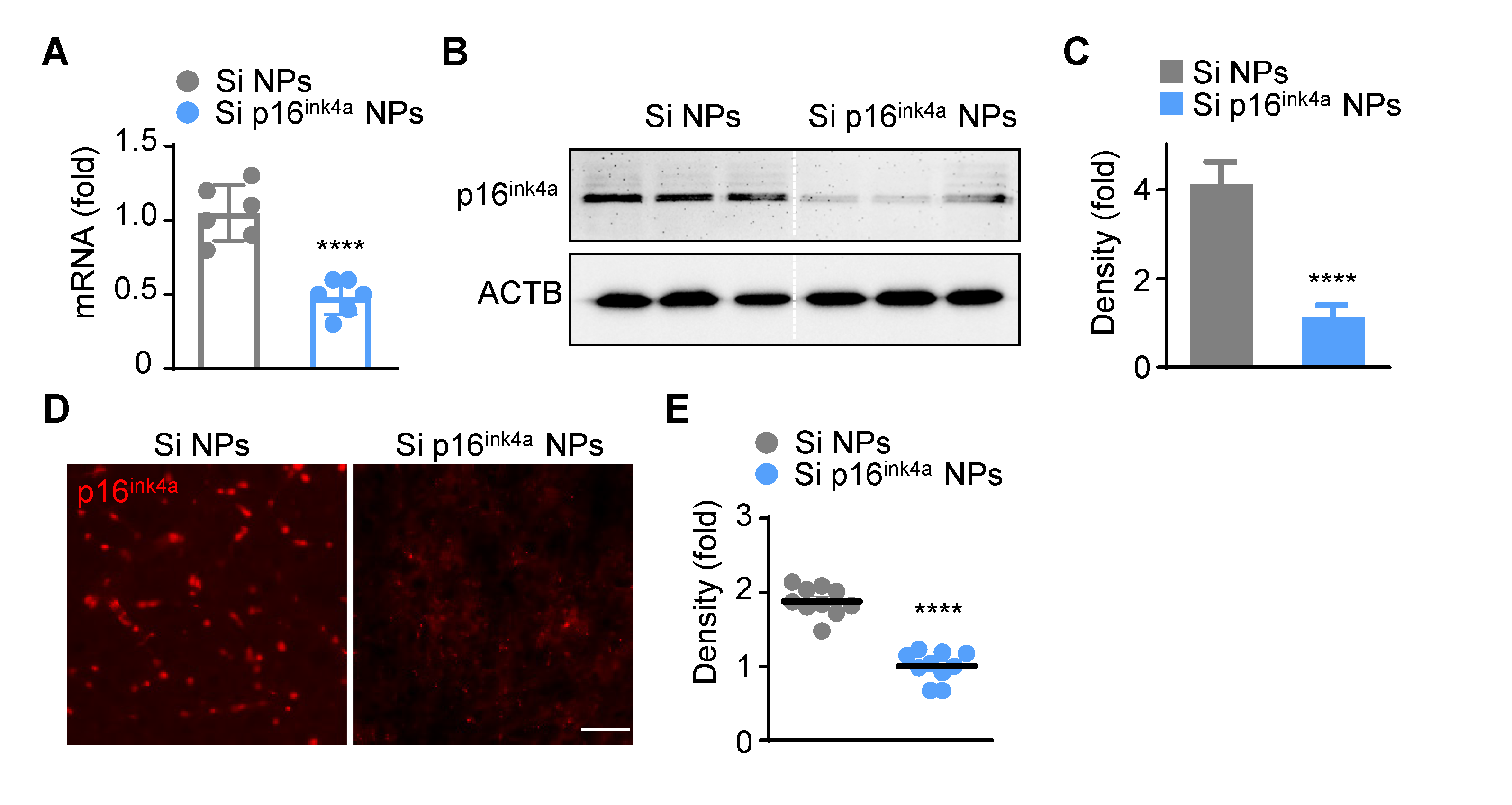

Supplement: Supplementary file 5 — Additional file 5: Supplementary Figure 5. Poly(D,L-lactic-co-glycolic acid) nanoparticles encapsulated with p16ink4a siRNA attenuated the expression of p16ink4a. (A) mRNA expression of p16ink4a was quantified by qPCR in the cortex of 4-month-old 5XFAD mice injected intrathecally into the cisterna magna with scrambled siRNA NPs or p16ink4a siRNA NPs once per week for 8 weeks. ****p < 0.001, versus mice treated with scrambled siRNA NPs; unpaired Student’s t test. (B) Western blot of p16ink4a expression in brain tissue of 4-month-old 5XFAD mice injected with scrambled siRNA NPs or p16ink4a siRNA NPs once per week for 8 weeks. (C) Quantification of (B). ACTB: β-actin. ****p< 0.001, versus mice treated with scrambled siRNA NPs; unpaired Student’s t test. (D) Immunostaining with anti-p16ink4a antibody of cortex brain sections from 8-month-old 5XFAD mice. Scale bar: 100 µm. (E) Quantification of p16ink4a expression in (C). ****p < 0.001, versus mice treated with scrambled siRNA NPs; unpaired Student’s t test. Si: siRNA. [file 13024_2024_715_MOESM5_ESM.tif]

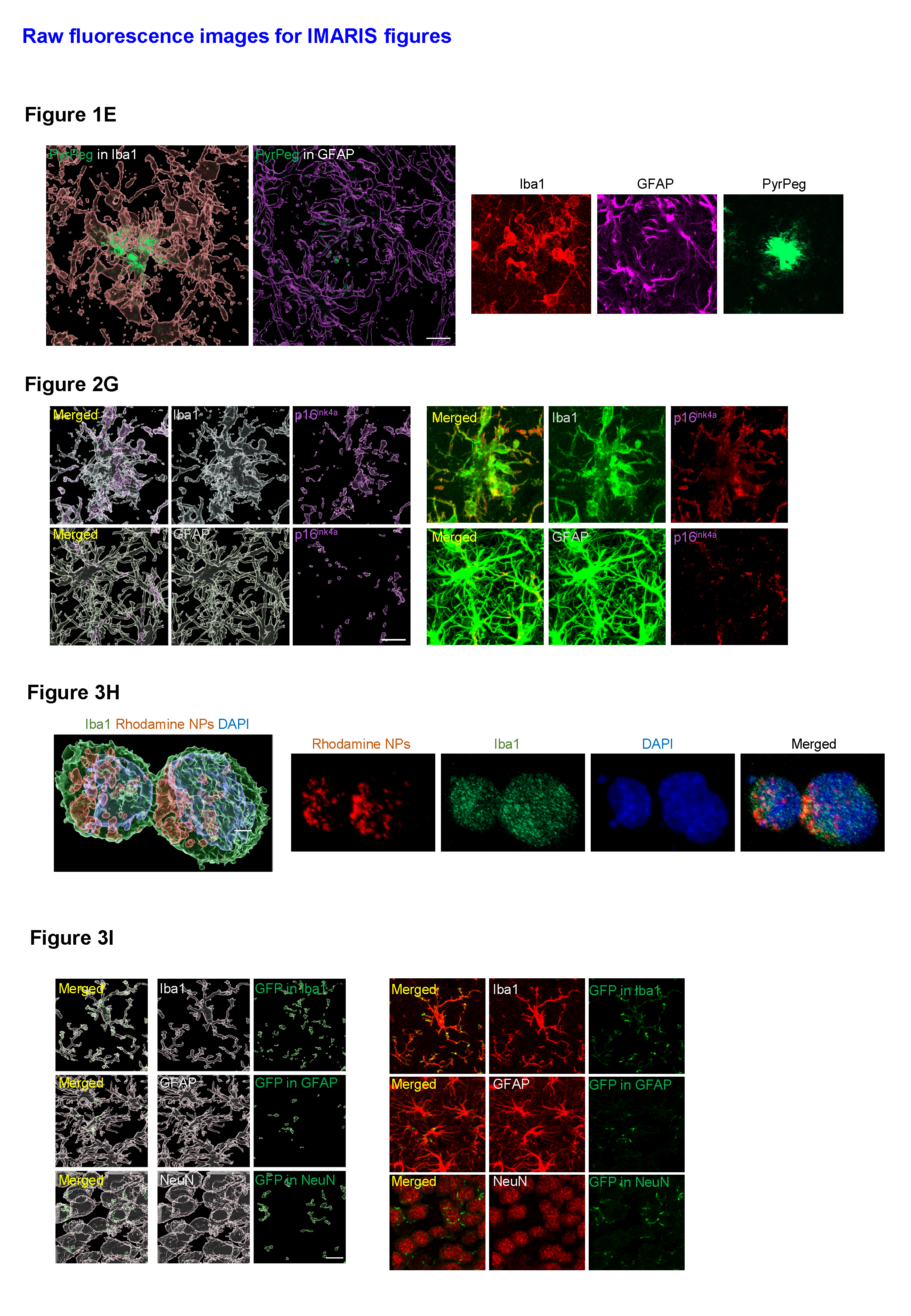

Supplement: Supplementary file 6 — Additional file 6: Supplementary Figure 6. Raw fluorescence images for IMARIS figures. [file 13024_2024_715_MOESM6_ESM.tif]

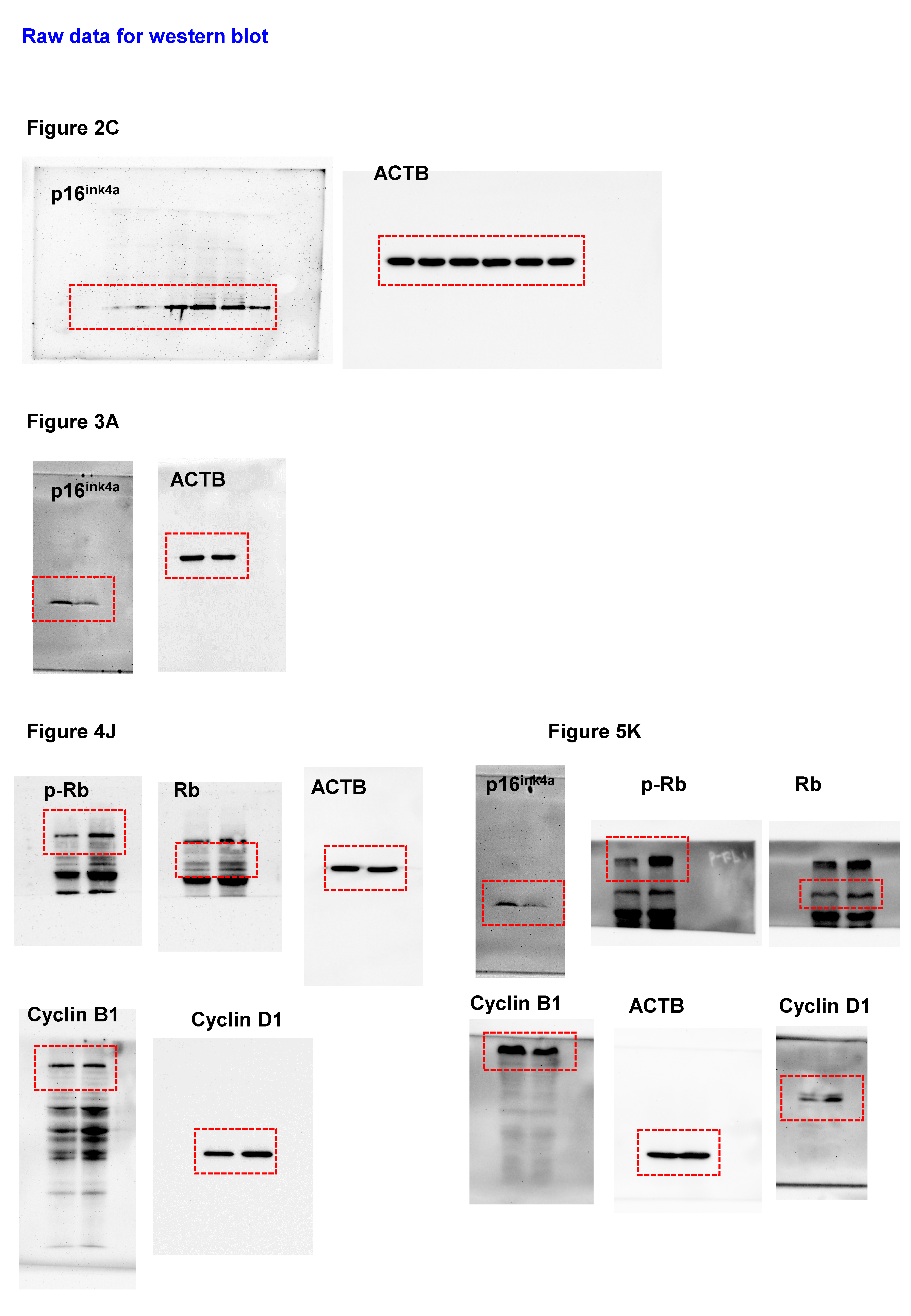

Supplement: Supplementary file 7 — Additional file 7: Supplementary Figure 7. Raw data for western blot. [file 13024_2024_715_MOESM7_ESM.zip › Suppl Figure 7-1.tif]

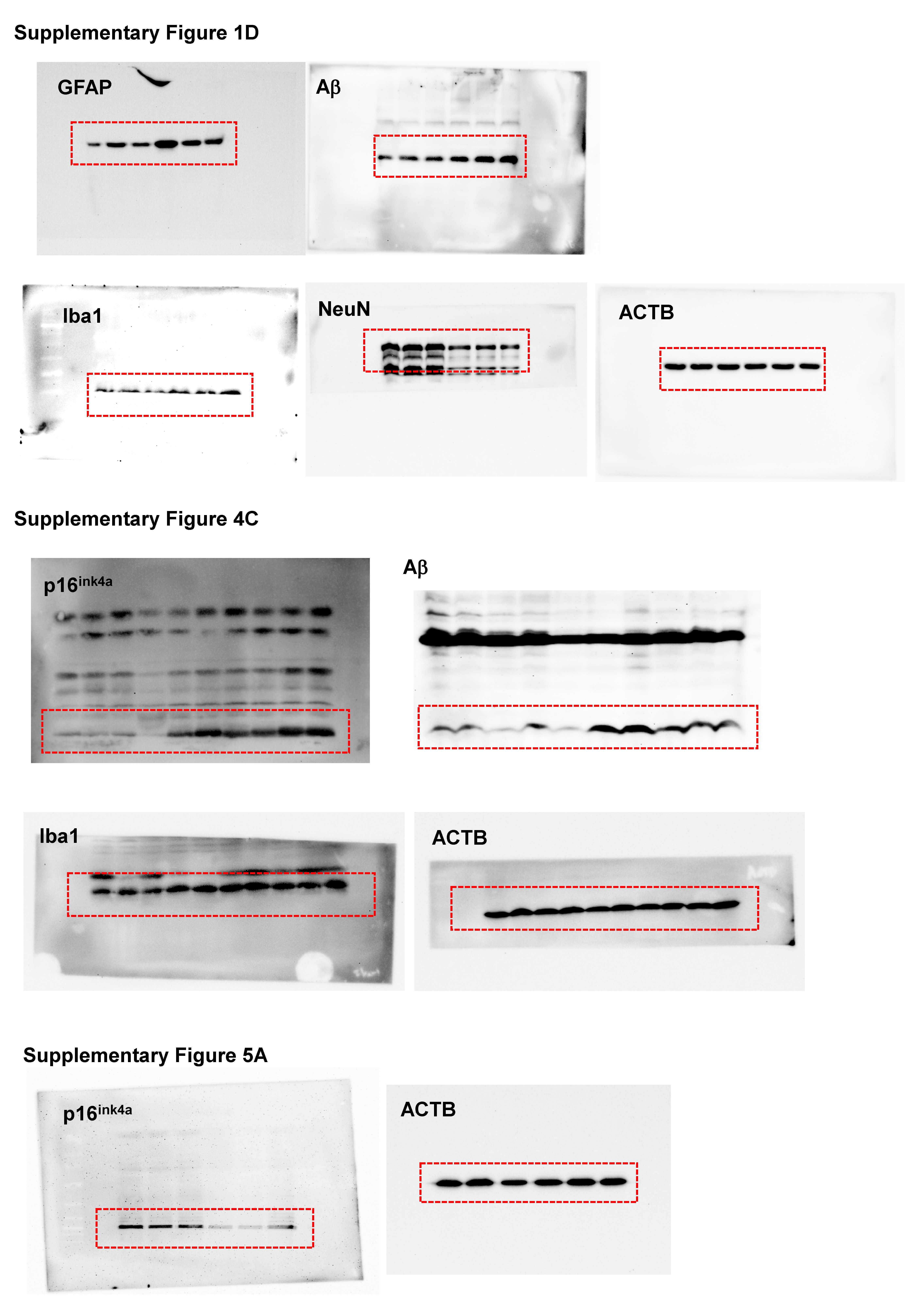

Supplement: Supplementary file 7 — Additional file 7: Supplementary Figure 7. Raw data for western blot. [file 13024_2024_715_MOESM7_ESM.zip › Suppl Figure 7-2.tif]
