# Supplementary material for: Rejuvenating aged microglia by p16ink4a-siRNA-loaded nanoparticles increases amyloid-β clearance in animal models of Alzheimer’s disease
Source: Mol Neurodegener. 2024 Mar 16;19:25. doi: 10.1186/s13024-024-00715-x (PMC10943801; doi:10.1186/s13024-024-00715-x)
Supplement: Supplementary file 8 — Additional file 8: Supplementary Table 1. Statistical analyses. [file 13024_2024_715_MOESM8_ESM.pdf]

	Figure number	Test	Technic		Treatment	Genotype	Sex	Age	Number	Mean	SEM(±)	Statistical test	Stat	P value	post hoc test	Comparison	t	P value
Figure	1C	co-localization test	Immnohistochemistry	co-local area with Aβ		5XFAD	M	8-9m	10 (for each groups)	-3.188	0.3148	unpaired t-test		<0.0001				
	1F	cell volume analysis	Immnohistochemistry	PyrPeg in cell unit		5XFAD	M	8-9m	10 (for each groups)	-297	30.91	unpaired t-test		<0.0001				
	2E	expression density	Immnohistochemistry	p16ink4a expression		5ZXFAD / age matched littermate control	M	8-9m	10 (for each groups)	3.614	0.8285	unpaired t-test		0.0005				
	2H	celll unit analysis	Immnohistochemistry	p16ink4a expression with Iba1 or GFAP		5ZXFAD / age matched littermate control	M	8-9m	10 (for each groups)	37.08	2.845	unpaired t-test		<0.0001				
	3A	expression density	western blot	$p16^{ink4a}$		BV2 cell				-1.5667	0.2963	unpaired t-test		0.0061				
	3B	expression density	qRT-PCR	$p16^{ink4a}$		BV2 cell				-0.8275	0.1126	unpaired t-test		0.0003				
	3F	cell viability test	MTT assay	$p16^{ink4a}$ PLGA NPs		BV2 cell						Two-way ANOVA	F(3, 15) = 2.083	0.6126	Bonferroni's test			
	3J	cell unit analysis	Immnohistochemistry	AAV-GFP NPs		WT	M	7w-8w	10 (for each groups)			one-way ANOVA		0.0115	tukey test	GFAP vs Iba1		<0.0001
																GFAP vs NeuN		0.0046
																Iba1 vs NeuN		<0.0001
	4B	distance/velocity	behavior test		$p16^{ink4a}$ PLGA NPs/scramble siRNA PLGA NPs	5XFAD	M	8-9m	7 (for each groups)			Two-way ANOVA	F(2, 39) = 0.07171	0.9309	Bonferroni's test	trial 1		0.4284
																trial 2		0.7481
																trial 3		0.5151
	4E	time to target	behavior test		$p16^{ink4a}$ PLGA NPs/scramble siRNA PLGA NPs	5XFAD	M	8-9m	8 (for each groups)			Two-way ANOVA	F(4, 56) = 13.74	<0.0001	Bonferroni's test	Trial 1		>0.9999
																Trial 2		<0.0001
																Trial 3		<0.0001
																Trial 4		<0.0001
																Trial 5		<0.0001
	4G	cell area analysis	Immnohistochemistry	Abeta	p16ink4a PLGA NPs/scramble siRNA PLGA	5XFAD	M	8-9m	7 (for each			one-way ANOVA	F(3, 24) = 63.49	<0.0001	tukey test	Cortex		<0.0001
																hippocampus		<0.0001
	4H	cell surface area	Immnohistochemistry	Iba1	p16ink4a PLGA NPs/scramble siRNA PLGA NPs	5XFAD	M	8-9m	10 (for each groups)	-1.78	0.1952	unpaired t-test		<0.0001				
		plaque-close cell number	Immnohistochemistry	Iba1	p16ink4a PLGA NPs/scramble siRNA PLGA NPs	5XFAD	M	8-9m	10 (for each groups)	-1.75	0.1561	unpaired t-test		<0.0001				
	4I	Abeta amount	ELISA	Abeta	p16ink4a PLGA NPs/scramble siRNA PLGA NPs	5XFAD	M	8-9m	8 (for each groups)	-37.57	2.415	unpaired t-test		<0.0001				
	4J	expression density	western blot	p-Rb/Rb,	p16ink4a PLGA NPs/scramble siRNA PLGA NPs	5XFAD	M	8-9m	9 (for each groups)	1.56	0.1939	unpaired t-test		<0.0001				
				Cyclin D1	p16ink4a PLGA NPs/scramble siRNA PLGA NPs	5XFAD	M	8-9m	9 (for each groups)	0.54	0.088	unpaired t-test		<0.0001				
				Cyclin B1	p16ink4a PLGA NPs/scramble siRNA PLGA NPs	5XFAD	M	8-9m	9 (for each groups)	0.55	0.1286	unpaired t-test		<0.0001				
	5B	staining density	SA-beta-gal assay	colocal correlation	p16ink4a PLGA NPs/scramble siRNA PLGA NPs	5XFAD	M	8-9m	6 (for each groups)	-0.1159	0.02198	unpaired t-test		<0.0001				
	5C	colocalization	SA-beta-gal assay	colocalization	p16ink4a PLGA NPs/scramble siRNA PLGA NPs	5XFAD	M	8-9m	6 (for each groups)	-3.792	0.4752	unpaired t-test		<0.0001				
	5D	expression fold	p16ink4a	mRNA	p16ink4a PLGA NPs/scramble siRNA PLGA NPs	5XFAD	M	8-9m	6 (for each groups)			Two-way ANOVA	F(10,110)=52.39	<0.0001	Tukey test			<0.0001
			p21															
			IL1a															
			IL!b															
			IL6															
			Mmp3															
			Mmp12															
			Ccl8															
			Cxcl1															
			Cxcl2															

			Timp1															
	5E	cell number counting	Immnohistochemistry	ki67	p16ink4a PLGA	5XFAD	M	8-9m	9 (for	1.911	0.1495	unpaired t-test		<0.0001				
	5F	cell number counting	Immnohistochemistry	Lamp1	p16ink4a PLGA	5XFAD	M	8-9m	9 (for	1.956	0.136	unpaired t-test		<0.0001				
	5I	cell number counting	Immnohistochemistry	TREM2	p16ink4a PLGA	5XFAD	M	8-9m	9 (for	1.978	0.1223	unpaired t-test		<0.0001				
	5J	cell number counting	Immnohistochemistry	clec7a	p16ink4a PLGA	5XFAD	M	8-9m	9 (for	1.733	0.1577	unpaired t-test		<0.0001				
	6B	phagocytosis analysis	Immnohistochemistry	phagosome number	p16ink4a siRNA/scramble siRNA	BV2 cell						Two-way ANOVA	F(2, 18) = 53.24	<0.0001	tukey test	<5		<0.0001
	6E	Density	FACS	phagosome density	p16ink4a siRNA/scramble siRNA	BV2 cell						Two-way ANOVA	F(4, 20) = 37.36	<0.0001	tukey test	2 hours<br>1 day		<0.0001<br><0.0001
	6J	cell unmber (%)	FACS	G2/M, S, G0/G1	p16ink4a siRNA/scramble siRNA	BV2 cell						Two-way ANOVA	F(2 ,18) = 6.256	0.0087	Bonferroni's test	G0/G1		0.0432
	6L	expression density	western blot	p-Rb/Rb, Cyclin D1, Cyclin B1	p16ink4a siRNA/scramble siRNA	BV2 cell				1.92<br>0.728<br>-0.672	0.2074<br>0.1338<br>0.1255	unpaired t-test		<0.0001<br>0.0006<br>0.0007				

	Figure number	Test	Technic		Treatment	Genotype	Sex	Age	Number	Mean	SEM(±)	Statistical test	Stat	P value	post hoc test	Comparison	t	P value
Supple	1E	expression density	western blot	GFAP		5XFAD, WT	M	8-9m	6	0.6625	0.2239	unpaired t-test		0.0253				
				Ab		5XFAD, WT	M	8-9m	6	1.033	0.3061	unpaired t-test		0.0331				
				NeuN		5XFAD, WT	M	8-9m	6	-0.4725	0.02869	unpaired t-test		0.0022				
				Iba1		5XFAD, WT	M	8-9m	6	0.94	0.2767	unpaired t-test		0.0146				
	3	log2(CDKN2A TPM+1)	GTEx (genotype-tissue expression)	Amygdala			M/F	by age	152			Kruska-wallis test			Dunn's multiple comparisons test	20-29 vs all		n.s.
				Anterior cingulate cortex					176							20-29 vs 30-39		0.05
				caudate basal ganglia					246							20-29 vs all		n.s.
				cerebellum					241							20-29 vs all		n.s.
				cortex					255							20-29 vs 60-69		0.0028
																20-29 vs 70-79		0.0075
				frontal cortex BA9					209							20-29 vs 60-69		0.034
				hippocampus					197							20-29 vs 60-69		0.0148
																20-29 vs 70-79		0.0157
				hypothalamus					202							20-29 vs all		n.s.
				nucleus aaaumbens basalganglia					246							20-29 vs all		n.s.
				putamen basal ganglia					205							20-29 vs all		n.s.
				spinal cord cervical C1					159							20-29 vs all		n.s.
				substantial nigra					139							20-29 vs all		n.s.
	4D	expression density	western blot	$p16^{ink4a}$		AD patient/healthy control	F/M		5	1.376	0.3067	unpaired t-test		0.002				
				Ab		AD patient/healthy control	F/M		5	1.336	0.3595	unpaired t-test		0.0059				
				Iba1		AD patient/healthy control	F/M		5	0.322	0.0823	unpaired t-test		0.0045				
	5A	expression	qRT-PCT	$p16^{ink4a}$	p16ink4a PLGA NPs/scramble siRNA PLGA NPs	5XFAD TG	M	8-9m	6	-0.5667	0.09006	unpaired t-test		<0.0001				
	5C	expression density	western blot	$p16^{ink4a}$	p16ink4a PLGA NPs/scramble siRNA PLGA NPs	5XFAD TG	M	8-9m	6	-3	0.2908	unpaired t-test		<0.0001				
	5E	expression density	Immnohistochemistry	$p16^{ink4a}$	p16ink4a PLGA NPs/scramble siRNA PLGA NPs	5XFAD TG	M	8-9m	10	-0.88	0.08943	unpaired t-test		<0.0001				
