# Supplementary material for: Rejuvenating aged microglia by p16ink4a-siRNA-loaded nanoparticles increases amyloid-β clearance in animal models of Alzheimer’s disease
Source: Mol Neurodegener. 2024 Mar 16;19:25. doi: 10.1186/s13024-024-00715-x (PMC10943801; doi:10.1186/s13024-024-00715-x)
Supplement: Supplementary file 9 — Additional file 9: Supplementary Table 2. Top 5 gene ontology terms involved with the 418 increased and 375 decreased differentially expressed genes in amyloid-β42-positive microglia from old mice compared with young mice. [file 13024_2024_715_MOESM9_ESM.pdf]

Up-regulation	Fold Enrichment	(-)LOG10(FDR)
**GO biological process complete**		
mitotic cell cycle (GO:0000278)	**6.82**	**25.10**
mitotic cell cycle process (GO:1903047)	**7.18**	**23.85**
cell cycle (GO:0007049)	**4.26**	**23.28**
cell cycle process (GO:0022402)	**5.16**	**21.11**
cell division (GO:0051301)	**6.05**	**17.73**
**GO cellular component complete**		
non-membrane-bounded organelle (GO:0043228)	**2.08**	**16.01**
intracellular non-membrane-bounded organelle (GO:0043232)	**2.08**	**15.71**
intracellular anatomical structure (GO:0005622)	**1.31**	**12.72**
chromosome (GO:0005694)	**3.15**	**11.64**
intracellular organelle (GO:0043229)	**1.35**	**11.62**
**GO molecular function complete**		
microtubule binding (GO:0008017)	**5.75**	**6.87**
tubulin binding (GO:0015631)	**4.37**	**5.58**
adenyl nucleotide binding (GO:0030554)	**2.36**	**5.06**
ATP binding (GO:0005524)	**2.41**	**5.04**
adenyl ribonucleotide binding (GO:0032559)	**2.38**	**5.04**
**Down-regulation**	**Fold Enrichment**	**(-)LOG10(FDR)**
**GO biological process complete**		
regulation of nitrogen compound metabolic process (GO:0051171)	**1.76**	**9.28**
negative regulation of biological process (GO:0048519)	**1.77**	**9.22**
negative regulation of cellular process (GO:0048523)	**1.83**	**9.07**
regulation of primary metabolic process (GO:0080090)	**1.72**	**8.92**
regulation of cellular metabolic process (GO:0031323)	**1.69**	**8.42**
**GO cellular component complete**		
intracellular organelle (GO:0043229)	**1.28**	**5.70**
nucleus (GO:0005634)	**1.48**	**5.68**
intracellular anatomical structure (GO:0005622)	**1.23**	**5.64**
organelle (GO:0043226)	**1.27**	**5.45**
RNA polymerase II transcription regulator complex (GO:0090575)	**4.92**	**4.64**
**GO molecular function complete**		
protein binding (GO:0005515)	**1.39**	**5.22**
transcription factor binding (GO:0008134)	**2.89**	**2.99**
binding (GO:0005488)	**1.2**	**2.83**
enzyme binding (GO:0019899)	**1.83**	**2.64**
organic cyclic compound binding (GO:0097159)	**1.48**	**2.64**
